# State of the Art of Cyclic Lipopeptide–Membrane Interactions: Pore Formation and Bilayer Permeability

**DOI:** 10.3390/pharmaceutics17091142

**Published:** 2025-08-31

**Authors:** Anastasiia A. Zakharova, Svetlana S. Efimova, Olga S. Ostroumova

**Affiliations:** Institute of Cytology of Russian Academy of Science, Tikhoretsky ave. 4, 194064 St. Petersburg, Russia

**Keywords:** lipid membrane, ion-permeable pores, cyclic lipopeptides, *Pseudomonas* spp., *Streptomyces* spp., *Bacillus* spp., cyanobacteria

## Abstract

**Background/Objectives**: Resistance of pathogenic microorganisms to antibiotics poses a serious threat to public health and often leads to devastating consequences. In this context, one of the pressing challenges in pharmacochemistry is the search for new, effective antibiotics to combat severe human diseases. Cyclic lipopeptides have emerged as some of the most promising candidates and have been widely studied. These compounds are a class of microbial secondary metabolites produced by various microorganisms, and they possess significant medical and biotechnological importance. The defining structural feature of these compounds is the presence of both a hydrophobic fragment, primarily a hydrocarbon tail of varying length, and a hydrophilic cyclic peptide moiety. This hydrocarbon tail confers amphiphilic properties to the lipopeptides, which are essential for their broad spectrum of biological activities. Their mechanism of action involves disruption of the cell membrane, and in many cases, the formation of ion-permeable defects has also been shown. **Results**: This review summarizes the data on cyclic lipopeptides produced by *Pseudomonas* spp., *Streptomyces* spp., and *Bacillus* spp. that modify membrane permeability through the formation of ion channels. The main emphasis is on understanding how the structure of the CLP can be related to the probability and mode of pore formation. **Conclusions**: The findings can contribute to expanding the arsenal of effective antimicrobial agents with a mechanism of action that reduces the risk of developing resistance.

## 1. Introduction

Natural antimicrobial cyclic lipopeptides (CLPs) serve as a primary defense mechanism for hosts against pathogens and play a crucial role in the innate immune system. Their widespread occurrence across diverse species underscores their fundamental importance in organismal defense. Natural lipopeptides are often considered superior to synthetic counterparts because they exhibit lower toxicity, better environmental compatibility, and greater biodegradability. These properties make natural lipopeptides safer and more sustainable alternatives to chemical surfactants and synthetic compounds. Additionally, they also demonstrate strong biological activity even at low doses, with minimal toxicity, and lower risks of adverse effects such as allergies and irritation. Due to these advantages, natural lipopeptides are favored in diverse applications, including pharmaceuticals, cosmetics, and agriculture.

The substantial structural diversity of CLPs produced by various microbial strains suggests a wide range of natural physiological functions [1,2]. For example, CLPs have been implicated in growth and differentiation processes, acting as signaling molecules [3,4,5]. However, their most clinically significant property is their antagonistic and antimicrobial activity. This antagonism confers a competitive advantage to the producing host by inhibiting or lysing other microorganisms, including fungi, viruses, bacteria, mycoplasmas, and oomycetes. While synthetic lipopeptides offer benefits like improved stability and tailored biological activity, natural lipopeptides provide crucial advantages such as broad-spectrum antimicrobial, anti-aging, and bio-control properties that arise from natural biological systems.

The antimicrobial activities of CLPs encompass antiviral, antibacterial, and antifungal effects, effectively suppressing many pathogenic strains responsible for widespread human diseases. For instance, Groupe et al. demonstrated that viscosin exhibits activity against enveloped viruses [6], while surfactin’s antiviral properties have also been documented [7]. Polymyxin B, one of the earliest clinically used CLPs, has regained importance due to the rise of multidrug-resistant Gram-negative bacteria, despite its known severe toxicity [8]. Its antibacterial spectrum includes many Gram-negative aerobic pathogens such as species of the *Enterobacteriaceae* family (*Escherichia coli*, *Enterobacter* spp., *Salmonella* spp., *Citrobacter* spp., *Klebsiella* spp., and *Shigella* spp.) [9,10,11,12,13,14]. Moreover, polymyxins are effective against non-enzymatic Gram-negative pathogens like *Acinetobacter baumannii* and *Pseudomonas aeruginosa*. Daptomycin, a calcium-dependent CLP synthesized from a derivative of compound A2l978Cs (LYI46032), is another important antimicrobial used to treat infections caused by Gram-positive bacteria, including *Staphylococcus aureus*, *Staphylococcus epidermidis*, *Streptococcus viridans*, *Streptococcus pneumoniae,* and *Enterococcus* spp. Similar calcium-dependent CLPs such as friulimicin B and amphomycin exhibit comparable spectra of activity [15,16]. Cyanobacteria are a rich and relatively new source of structurally diverse CLPs, as highlighted by several studies [17,18,19,20,21]. Many cyanobacterial lipopeptides have already been tested in disease models and demonstrate promising potential as drug candidates. These compounds are particularly relevant for pharmaceutical development, with applications spanning anticancer, antiviral, antifungal, and anti-inflammatory therapies [22]. Furthermore, they hold promise for treating diseases such as cancer, parasitic infections, and viral diseases including HIV and COVID-19 [23].

**Figure 1:**
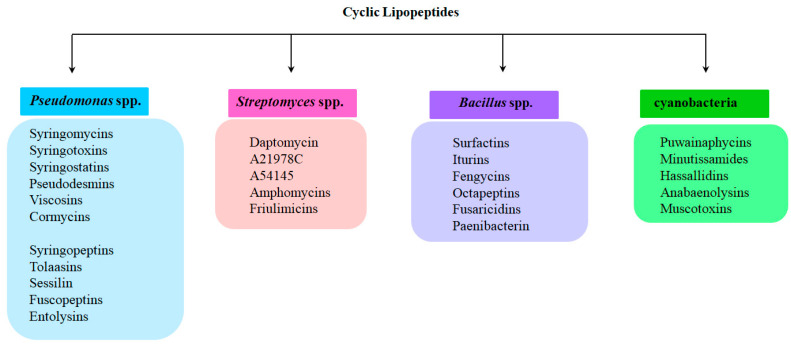
Schematic representation of the classification of cyclic lipopeptides (CLPs) with examples of representatives in each group exhibiting membrane activity.

This review will focus on major common CLP producers, including *Pseudomonas* spp., *Streptomyces* spp., *Bacillus* spp., and cyanobacteria (Figure 1). Most CLPs possess amphipathic properties, enabling interactions with lipid membranes to alter membrane permeability, often through pore formation. Table 1 summarizes selected CLPs, their putative lipid targets in model membranes, and the CLP threshold concentration required to observe ion-permeable pores. However, only a limited number have been characterized in detail, as discussed below.

## 2. Cyclic Lipopeptides Produced by *Pseudomonas* spp.

**Figure 2:**
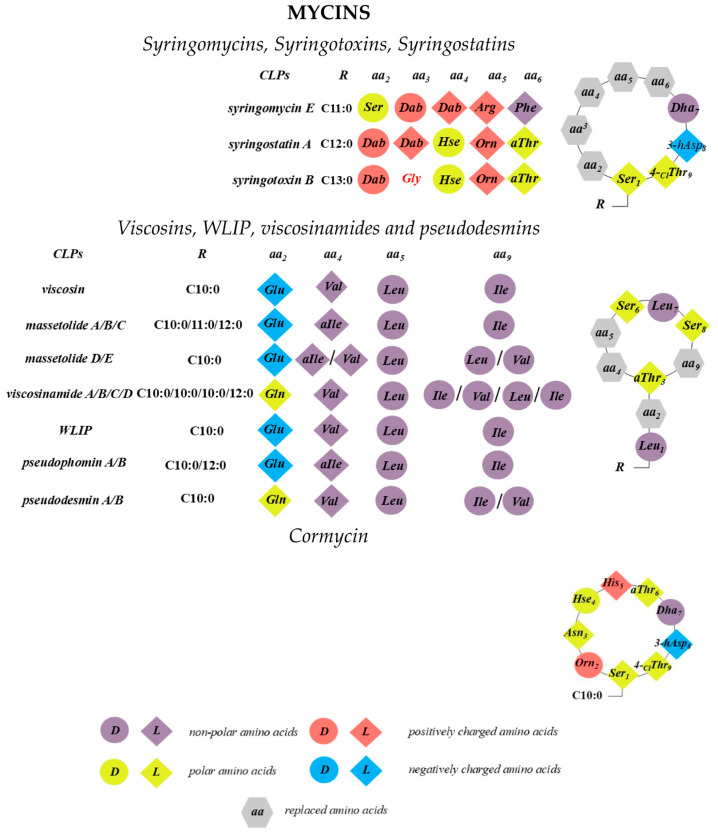
Chemical structures of pore-forming CLPs produced by *Pseudomonas* spp. (subclass mycins). Abbreviations: *Dab*—2,4-diaminobutyric acid; *Hse*—homoserine; *Orn*—ornithine; *aThr—allo* threonine; *3-hAsp*—3-hydroxy aspartic acid; *4-_Cl_Thr*—4-chlorothreonine.

**Figure 3:**
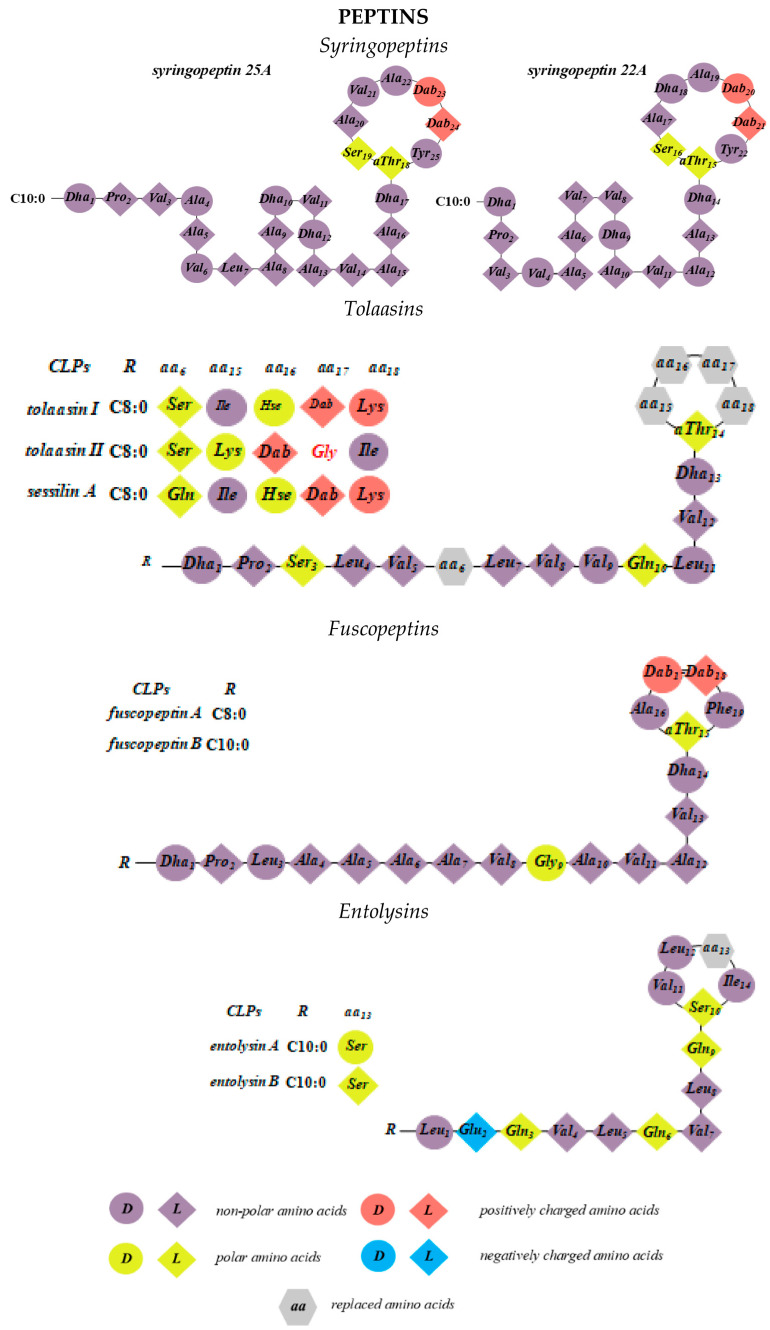
Chemical structures of pore-forming CLPs produced by *Pseudomonas* spp. (subclass peptins). Abbreviations: *Dab*—2,4-diaminobutyric acid; *Hse*—homoserine; *Orn*—ornithine; *aThr—allo* threonine; *Dha*—2,3-dehydro-2-aminobutyric acid; *3-hAsp*—3-hydroxy aspartic acid; *4-_Cl_Thr*—4-chlorothreonine.

Sinden and DeVay showed that isolates of Gram-negative bacteria of *Pseudomonas* spp. from stone fruit trees produce a substance in culture that is toxic to peach trees as well as to various fungi and bacteria [58]. Moreover, the pathogenicity of these bacteria correlates with their ability to produce this toxin. It was subsequently demonstrated that, in particular, the capacity of *Pseudomonas syringae* pv. *syringae* to cause necrotic infections in a wide range of plants is linked to its production of two types of lipodepsipeptide phytotoxins containing unusual amino acids with d-stereochemistry [59,60,61,62,63]. The first group includes nonapeptides with a cyclic head comprising 9–10 amino acid residues, including syringomycins, syringotoxins, syringostatins, viscosins, WLIP, viscosinamides, pseudomycins, and cormycin (Figure 2). The second group consists of larger molecules containing 18 to 25 amino acid residues, most with d-stereochemistry; representatives include syringopeptins, tolaasins, fuscopeptins, and entolysins [64] (Figure 3). In this latter subclass, the C-terminal fragment forms a lactone ring of five (tolaasins and fuscopeptins) to eight (syringopeptins) amino acids [2,65,66].

The oligopeptide moiety may be composed of a combination of standard proteinogenic amino acids, modified amino acids, and non-proteinogenic amino acids. These can include dehydrated amino acids such as 2,3-dehydroaminobutyric acid, chlorinated amino acids like 4-chloro-threonine, d-allo-configured amino acids, acylated forms such as N5-acetyl-N5-hydroxy-ornithine, and hydroxylated amino acids including 3-hydroxyaspartic acid or the rare α-hydroxy-ornithine (Figure 2). Additionally, biosynthetic intermediates of proteinogenic amino acids, such as *Orn*, *Hse*, and *Dab*, may be incorporated into the peptide chain [67]. Despite their structural diversity, pseudomonal CLPs, with the exception of the syringomycin-like group [68,69,70,71,72,73,74,75], share a common feature: at least half of their amino acids are hydrophobic residues such as *Ala*, *Val*, *Leu*, *Ile*, and *Phe*. Short CLPs from *Pseudomonas* spp. typically share several key characteristics: (1) a macrolactone ring formed through a bond between the C-terminal end of the peptide and the hydroxyl group of a *Ser* or *Thr* residue; (2) a conserved *N*-terminal motif comprising a *Leu* linked to one of the hydrophilic amino acids *Glu*, *Gln*, or *Asp*; and (3) an overall prevalence of acidic amino acids in their chemical structures, with the exception of syringomycin-like CLPs, ferrocins, and pseudofactins [76,77,78]. The structural diversity among these groups mainly arises from variations in fatty acid chains, configurational inversion of specific amino acids, and substitution among chemically similar residues (e.g., *Glu/Gln* or *Val/Leu/Ile*).

Most CLPs produced by *Pseudomonas* spp. exhibit broad-spectrum antifungal activity [79]. For example, the CLP massetolide A, produced by various *Pseudomonas* strains, has been shown to enhance tomato resistance against *Phytophthora* infestans infection [80]. Similarly, orfamide and sessilin, produced by *Pseudomonas* spp. CMR12a, induce systemic resistance in rice and beans [81]. Among well-known CLPs from this group, syringomycin E and syringopeptin demonstrate high efficacy against diseases affecting economically important crops. Syringomycin E has been shown to suppress the growth of green mold pathogens such as *Penicillium digitatum*, *Rhodotorula pilimanae*, and *Geotrichum citriaurantii* [82]. Kawasaki et al. [83] explored the potential use of syringomycins as agrofungicides and seed treatments against the soil-borne oomycete *Pythium ultimum*. These CLPs also strongly inhibit the growth of *Botrytis cinerea*, a mold responsible for gray mold disease in many plants [84], and *Venturia inaequalis*, the causative agent of apple scab [85]. Fuscopeptins, structurally related to syringopeptins, exhibit biological activity including damage to plant tissues and antifungal effects against *Geotrichum candidum* and *Saccharomyces cerevisiae* [86]. Additional examples include tolaazine D, which inhibits growth of the phytopathogens *Rhizoctonia solani* and *Rhodococcus fascians* [87]; pseudophomines A and B, which significantly suppress *Alternaria brassicae*, *Phoma lingam*, and *Sclerotinia sclerotiorum* [88]; and amphisin, loquisin, tensin, and viscosinamide, all of which demonstrate antagonistic activity against *P. ultimum* and *R. solani* [89,90].

### 2.1. Syringomycins, Syringotoxins, Syringostatins

*Pseudomonas syringae* pv. *syringae* is a phytopathogenic bacterium causing necrotic infections across a wide range of plants [59,60,61,62,63,64]. Among these, syringomycins are mainly associated with the pathogen’s virulence. Syringomycin-like CLPs contain *Dha, Dab*, and a crucial C-terminal 4-chlorothreonine (*Thr[4-Cl]*), which is important for antifungal activity [91]. A lactone ring is formed through the *N*-terminal *Ser* and this C-terminal *Thr[4-Cl]*. The fatty acid tail consists of 3-hydroxy or 3,4-dihydroxy fatty acids with 10 to 14 carbons (Figure 2). Three syringomycin variants (A1, E, and G) differ mainly in the fatty acid chain length [64,92].

Functional studies [93] showed that syringomycin inhibits growth of *S. cerevisiae* strains by altering plasma membrane electrical potential and proton efflux, indicating increased membrane permeability. Similarly, syringomycin treatment causes massive plasma membrane disruption in *Geotrichum candidum* [59]. Localization studies using fluorescent antibodies revealed that syringomycin concentrates around cell peripheries in phloem, xylem, cambium, and pith-parenchyma cells, supporting membrane localization [94]. Syringomycin induces alterations in ion transport, including K^+^ efflux via activation of membrane protein transporters, possibly mediated by ATPase phosphorylation [95,96], observations also confirmed in yeast models [93]. Other syringomycin-like toxins, such as syringostatin and syringotoxin, inhibit plasma membrane H^+^-ATPase activity in mung bean cells, possibly via detergent-like membrane disruption [97]. Syringotoxin decreases mitochondrial membrane potential and causes swelling by disturbing membrane permeability, likely due to K^+^ accumulation [98]. Syringomycin also induces Ca^2+^ leakage in beet storage tissue membranes [99].

A nonspecific effect of these CLPs on cell membranes is also evidenced by their hemolytic activity against red blood cells, which are not the biological target cells [27,28]. Syringomycins E and G, syringotoxin, and pseudomycins A, B, C, and C’ exhibit hemolytic activity on human red blood cells, though to varying extents [27]. The dose-dependence curves for hemolysis induced by syringomycins, syringotoxin, and pseudomycin are sigmoidal, indicating highly cooperative behavior and suggesting oligomerization of these CLPs [27]. Szabo et al. [31] further demonstrated that ion-permeable structures formed by syringotoxin specifically can increase membrane permeability to ^86^Rb^+^ and hemoglobin in a concentration-dependent manner.

All studied CLPs were able to interact with model lipid membranes [27,28]. Syringomycins, syringotoxin B, and pseudomycin induced leakage of calcein from liposomes composed of purified lipids. Importantly, the membrane permeabilization increased with the acyl chain length of the fatty acid moiety in the CLPs, as well as with the net positive charge component of the peptide [27]. For example, syringomycins carrying two positive charges exhibited substantially higher activity compared to pseudomycins with one charge, and syringomycin G (with a 3-hydroxytetradecanoic fatty acid) was more active than syringomycin E (with a 3-hydroxydodecanoic acyl chain).

Subsequent studies showed that almost all CLPs are capable of forming clearly detectable pores in planar model lipid membranes. The pore-forming activity of syringomycin E is the most thoroughly documented. Hutchison et al. [24] observed a step-like increase in the conductivity of model lipid membranes composed of phosphatidylethanolamine, phosphatidylserine, phosphatidylcholine, and cholesterol upon addition of syringomycin E at concentrations from 0.4 to 1.6 µM [24]. Single-channel current fluctuations were approximately 1 pA at +80 mV (Table 1). Similarly, Feigin et al. [25] reported that the one-sided addition of syringomycin E (0.8 to 4.1 µM) to PS lipid bilayers caused a voltage-dependent increase in membrane conductance, with single-pore currents around 0.7 pA at 100 mV (Table 1). These findings corroborate the measurements reported by Hutchison et al. [24].

Shchagina et al. [100,101] demonstrated that the pore-forming ability of syringomycin E is characterized by two stable conductivity states differing approximately 6-fold in magnitude, referred to as the “large” and “small” channels. Both channel types exhibit predominantly anion selectivity. The similar cation–anion selectivity and multiple conductivity levels of the large and small syringomycin E channels led the authors to propose that the large channels arise from the synchronous opening and closing of multiple elementary channels [102]. Ostroumova et al. [103], using polyethylene glycols of varying molecular weights, estimated that the channel openings on the cis- and trans-sides have radii of approximately 0.25–0.35 nm and 0.5–0.9 nm, respectively. The observed channel asymmetry may also result from an uneven distribution of syringomycin E molecules across the membrane, causing asymmetric current–voltage characteristics. Malev et al. [104] reported such asymmetric current responses upon application of external potentials of opposite polarity. The amplitude of syringomycin E channel currents strongly depends on the membrane lipid charge [104]. Specifically, conductance in neutral lipid membranes is about three times higher than in negatively charged bilayers.

These observations suggest that the membrane surface charge arising from charged lipid molecules and syringomycin E is likely responsible for the observed asymmetry in the current–voltage curves of single syringomycin E channels [104]. This hypothesis was later confirmed by Gurnev et al. [105]. Moreover, the influence of lipid composition on channel gating and charge distribution has been further elucidated in subsequent studies [105,106,107]. Channel formation in phosphatidylethanolamine membranes, which form non-lamellar inverted hexagonal phases in aqueous environments, requires approximately 15-fold higher concentration of syringomycin E compared to PC bilayers [106]. Analysis of the dependence of bilayer conductance on the concentration of CLP led to the conclusion that at least six syringomycin E molecules are necessary to assemble a functional conducting pore within the membrane [106]. This is consistent with observations that only monomeric antibiotic particles are present in aqueous solution [28]. Thus, syringomycin E channels are asymmetric peptide–lipid pores with a conical geometry, where the pore radii at the peptide and lipid interfaces are approximately 0.3 nm and 0.7 nm, respectively.

Gurnev et al. [26] demonstrated that channels formed by syringotoxin B and syringostatin A share characteristics similar to those of syringomycin E. Like syringomycin E, these CLPs induce both “large” and “small” ion channels in lipid bilayers. However, they differ in minimum pore-forming concentration, single-channel conductance, and clustering degree (the ratio between conductance of small and large channels). It was shown that the presence of 7.9–14.1 μM syringotoxin B or 1.4–2.1 μM syringostatin A induced step-like current fluctuations with amplitudes of approximately 16 pS and 18 pS, respectively, in phosphatidylcholine membranes [26]. Earlier, Ziegler et al. [108] reported that even lower CLP concentrations (0.7 μM) caused ion channel formation in bilayers composed of phospholipids isolated from soybeans, with step-like current amplitudes around 2 pA at 10 mV. Syringotoxin B incorporation into lipid membranes and pore formation was found to be about 10-fold more efficient at positive potentials than at negative ones. Similar to syringomycin E, application of a negative potential to membranes treated with syringotoxin B and syringostatin A induced channel closing [26]. The single-channel conductance of CLP-formed pores exhibited nonlinear and asymmetric dependence on transmembrane voltage, varying with the polarity of the applied potential.

Ziegler et al. [108], in their analysis of current–voltage relationships at salt gradients, revealed a chloride-to-potassium selectivity ratio of approximately 8:1 and a reversal potential near −47 mV, indicating that syringotoxin B channels are anion-selective. They proposed that these channels are formed not by individual syringotoxin B molecules, but by oligomeric complexes of a defined number of CLP molecules [30]. The maximum number of cooperatively functioning elementary channels is about seven for syringomycin E and between four and five for syringotoxin B and syringostatin A [31,32]. Differences in channel cluster properties may be attributed to the localization and number of charged *Dab* residues within the peptide ring of syringomycin-like CLPs [26]. Channels formed by syringotoxin B and syringostatin A displayed conductances approximately 10–30% higher than those formed by syringomycin E. Consistent with syringomycin E, negative potentials induced channel closure in membranes treated with these CLPs [26]. Szabo et al. [31] additionally determined that syringotoxin B pores are oligomers composed of 2 to 6 monomers. These findings suggest that syringotoxin B, syringostatin A, and syringomycin E share a similar pore-formation mechanism, involving oligomerization and voltage-dependent gating.

### 2.2. Viscosins, WLIP, Viscosinamides, and Pseudodesmins

Viscosins are a group of CLPs produced by *Pseudomonas* spp. and are considered one of the most extensive CLP families. This group includes the White Line Inducing Principle (WLIP) [109], massetolides [110], viscosinamides [111,112], pseudodesmins [113,114], and pseudophomins [115]. The primary microbial producers of different viscosins are *P. putida* and *Pseudomonas fluorescens*. These CLPs display a broad spectrum of biological activities, including antifungal, antibacterial, antiviral, and insecticidal effects, among others [116]. Structurally, the moiety of viscosin-group CLPs consists of a nine-amino-acid cyclic peptide ring formed by an ester bond between the C-terminal carbonyl and the side chain of a *Thr* residue at position 3 (*Thr_3_*) (Figure 2). The *N*-terminus is linked to a 3-hydroxy fatty acid, most commonly 3R-hydroxydecanoic acid. Structural variations within the viscosin group arise primarily from the identity of the amino acid at position 2, which can be either *Glu*—found in viscosin, WLIP, and massetolides—or *Gln*, typical for viscosinamides and pseudodesmins [111]. Another important variation is the stereochemistry at position 5, occupied by Leu, which can exist as either the l- or d-epimer. d-epimers include WLIP and pseudodesmins, whereas l-epimers are found in viscosin, viscosinamides, and massetolides. Additional differences stem from substitutions of hydrophobic residues at specific positions, likely due to substrate flexibility within the adenylation domains of nonribosomal peptide synthetases. In particular, residues at positions 4 and 9—typically *Ile*, *Leu*, or *Val*—can interchange. Likewise, the fatty acid chain length varies between C10 and C12. This variation can be observed even within the same bacterial strain, although predominant CLP profiles often vary among strains.

The molecular mechanism underlying the interaction of viscosin-group CLPs with target cells remains incompletely understood, but increased membrane permeability is recognized as a key factor. Thrane et al. [117] observed swelling of fungal hyphae of the plant pathogens *P. ultimum* and *R. solani* upon treatment with viscosinamide, suggesting that the swelling resulted from viscosinamide-induced ion channel formation leading to increased Ca^2+^ influx. Khattari et al. [118] demonstrated that viscosin’s disruptive effects on cells are predominantly driven by interactions with phospholipids. Incorporation of viscosin into DPPC monolayers altered Langmuir interfacial isotherms, indicating a concentration-dependent phase transition from liquid-ordered to liquid-disordered states. Geudens et al. [33] showed that viscosinamide, viscosin, and pseudodesmin interact similarly with small unilamellar vesicles composed of PG, PE, and CL (7:2:1 ratio), inducing comparable release of a fluorescent dye. The dose-dependence of dye release did not significantly differ between pseudodesmin A and viscosinamide A, nor between WLIP and viscosin, suggesting that subtle structural differences among these CLPs do not markedly impact their permeabilizing capacity. A detailed characterization of WLIP’s membrane interactions was performed using erythrocytes and artificial membranes [35,119]. Lo Cantore et al. [35] reported that WLIP induces erythrocyte lysis at minimal hemolytic concentrations around 2.7 μM. The hemolytic activity is caused by colloid-osmotic shock resulting from the formation of transmembrane pores. This effect can be prevented by the presence of osmoticants in the external medium, which counterbalance the osmotic imbalance. Kinetic analyses of hemolysis using polyethylene glycols of varying molecular weights estimated the functional pore radius of WLIP to be approximately 1.5 ± 0.1 to 1.7 ± 0.1 nm [35]. Given the small size of individual CLP monomers, these pores are likely aggregates of several monomers, consistent with a Hill coefficient of 8 ± 2 [35].

Sinnaeve et al. [113,120] proposed a model of the supramolecular self-association and pore-formation potency of viscosin-group CLPs using pseudodesmin A as a model. NMR spectroscopy revealed a two-step assembly process: initially, peptide monomers aggregate via hydrophilic side-chain interactions, forming disc-like structures. Subsequently, electrostatic interactions between oppositely charged oligopeptide termini link these discs into cylindrical supramolecular assemblies with hydrophilic interiors and hydrophobic exteriors. The size of these assemblies increases unidirectionally with rising pseudodesmin A concentration. Such supramolecular structures are hypothesized to insert into membranes to form ion-permeable transmembrane pores facilitating ion transport. An alternative mode of action involves CLP-induced asymmetry stress resulting in curvature strain and formation of toroidal or quasi-toroidal defects (pores) within the lipid bilayer. More recently, Steigenberger et al. [34] supported this hypothesis by showing calcein leakage from anionic liposomes composed of POPG and POPE, but not from POPC liposomes, upon treatment with viscosin and pseudodesmin A. This effect was attributed to repulsion between POPG and the CLPs, generating asymmetry stress between bilayer leaflets. The pore-forming efficacy of viscosin-group CLPs depends markedly on membrane lipid composition. For example, dye release induced by WLIP correlates positively with sphingomyelin content [35]. In contrast, increased sterol content decreases fluorescent probe leakage, a phenomenon similarly observed for syringomycin-group CLPs [27]. Geudens et al. [33] explained that sterols reduce the depth of CLP insertion into the bilayer, consequently diminishing their permeabilizing activity.

### 2.3. Cormycins

Cormycin A is a CLP produced by the pathogen *P. corrugata*. Its peptide moiety was elucidated by mass spectrometry and two-dimensional NMR methods, revealing a sequence of nine amino acid residues cyclized through a macrocyclic ring formed between the terminal carboxy group and the hydroxy group of the *N*-terminal *Ser* residue [36] (Figure 2). The hydrophobic moiety of cormycin A is represented by a 3,4-dihydroxyhexadecanoate fatty acid chain. In aqueous solution, cormycin A adopts a compact conformation characterized by an inward orientation of certain amino acid side chains and a “hairpin-bent” shape of the lipid moiety, stabilized by inter-residue interactions involving the peptide’s terminal region [36]. Scaloni et al. [36] reported that purified cormycin A inhibited bacterial growth of *Bacillus megaterium* and *R. pilimanae* at low micromolar concentrations, demonstrating higher antimicrobial activity compared to other CLPs derived from *P. syringae*. Cormycin A exhibited phytotoxic effects, as evidenced by chlorosis induction on *Nicotiana tabacum* leaves. The similarity in necrotic activity between the isolated CLP and culture filtrates of *P. corrugata* confirmed the essential role of cormycin A in the biological activity of this pathogen [121]. Hemolytic activity assays conducted by Scaloni et al. [36] showed that cormycin A lysed erythrocytes of human, rabbit, and sheep origin more effectively than syringomycin E and syringotoxin [28], suggesting that the lipid membrane is a primary biological target of cormycin A. Planar lipid bilayer experiments provided further support for a pore-forming mechanism of action. The addition of 11 µM cormycin A to the aqueous phase bathing a planar lipid bilayer composed of DPhPC and cholesterol at a 7:3 molar ratio induced discrete step-like increases in ionic current across the membrane (Table 1). Scaloni et al. [36] interpreted these current fluctuations as the formation of structured, ion-conducting channels rather than nonspecific detergent-like membrane disruption. Each step represented the activation of an individual channel with an average conductance of 3.9 ± 0.6 pS. Calcein leakage assays demonstrated that cormycin A’s permeabilizing activity was enhanced in liposomes containing sterols. Among tested sterols, the sensitivity of cormycin A followed the order: cholesterol > ergosterol > stigmasterol. However, the estimated number of cormycin A monomers constituting a functional ion-permeable channel was approximately 7 ± 1, independent of the sterol composition of the bilayer.

### 2.4. Syringopeptins

Syringopeptins 22A, 22B, 25A, and 25B were first described by Ballio et al. [61] and isolated from various plant tissues. The main producers of syringopeptins are strains of *P. syringae*, although they have also been found in cultures of *P. syringae* pv. *atrofaciens* and *P. syringae* pv. *lachrymans* [61,122,123]. Syringopeptin 22 homologs are produced by a *P. syringae* strain isolated from pear (B301) [61], as well as by strains isolated from sugarcane [124] and bean plants [125]. Syringopeptin 25 variants are produced by *P. syringae* strains from infected millet (B359) [61], citrus (B427) [61], wheat (M1) [126], and the wheat pathogen *P. atrofaciens* [122]. An isoform with a different C-terminal residue, syringopeptin 25-*Phe*, was reported in a laurel-infecting strain [127]. The secretion of syringopeptins by *Pseudomonas* spp. is typically accompanied by smaller cyclic nonapeptides, such as syringomycin [69,71,128], syringotoxin [72,129], syringostatin [71], or pseudomycin [68,130].

Syringopeptins 22 and 25 consist of 22 and 25 amino acids, respectively. Their sequences are predominantly hydrophobic, with valine and alanine residues being abundant. Most chiral centers adopt the d-configuration, including four *α*, *β*-unsaturated and two *Dab* acid residues [61,124,125,127]. Eight residues (from position 15 to 22 in syringopeptin 22, and 18 to 25 in syringopeptin 25) form a lactone ring via an ester linkage between the hydroxyl group of *allo*-threonine (*allo*-*Thr*) and the C-terminal *Tyr*. An *N*-terminal unusual 2,3-dehydro-2-aminobutyric acid is acylated with 3-hydroxydecanoic acid (A form) or 3-hydroxydodecanoic acid (B form). The net charge of syringopeptins 22 and 25 is +2. Ballio et al. [131] characterized the three-dimensional structure of syringopeptin 25A by two-dimensional NMR spectroscopy, revealing three main structural fragments: a loop comprising *Pro_2_* to *Val_6_*, an α-helical region (*Ala_8_* to *Ala_15_*), and a large lactone ring (*Thr_18_* to *Tyr_25_*).

Functionally, syringopeptin 25A, at concentrations ranging from 2 to 8 μM, induces electrolyte leakage in plant cells, leading to necrotic symptoms similar to those caused by syringomycin and syringostatin [62]. Lavermicocca et al. [84] showed that syringopeptin 25A causes more pronounced plasmolysis in potato tuber disks compared to syringopeptin 22A, inducing irreversible damage of approximately 40 and 30% after 1 h, respectively. Tobacco protoplasts and isolated maize roots were also highly sensitive, with about 50% lysis after 30 minutes’ exposure to 100 ng/mL of syringopeptins 22A and 22B [27,39]. Using the acidotropic dye neutral red, these authors demonstrated vacuolar content release induced by syringopeptin 25A, occurring via an “all-or-nothing” mechanism, likely limited by plasma membrane integrity loss that permits access to the vacuolar membrane.

Electrolyte leakage from plant tissues treated with syringopeptins was accompanied by net K^+^ efflux and Ca^2+^ influx in *S. cerevisiae* strain KZ1-1C whole cells [38]. Di Giorgio et al. [132] observed that syringopeptin promotes stomatal closure in *Xanthium strumarium* and *Vicia faba* L., potentially mediated by rapid K^+^ efflux, analogous to syringomycin’s effects [133]. Furthermore, syringopeptin 22A increased membrane permeability to ^86^Rb in red blood cells in a dose-dependent manner, with the sigmoidal efflux curve suggesting formation of ion-conducting clusters [37].

In artificial lipid systems, syringopeptins interact with lipid vesicles and planar bilayers similarly to syringomycin-like CLPs. They induce proton permeability changes in liposomes composed of PC and PE at nanomolar concentrations, which become dramatic at micromolar levels, collapsing the proton gradient [134]. Molecular dynamics simulations suggest that syringopeptins reduce lipid bilayer fluidity along lipid molecules’ entire length, indicative of pore formation [135].

Several studies (Table 1) confirm that syringopeptins 22 and 25 form single ion channels in planar lipid bilayers. Hutchison et al. [39] showed that syringopeptins 22A and 22B significantly reduce surface tension of phosphatidylethanolamine monolayers and form ion-permeable pores with picoampere-range currents in corresponding bilayers. Agner et al. [37] and Dalla Serra et al. [28] reported pores formed by syringopeptins 22A and 25A in lipid membranes composed of PE and PS or PC/PE/PS mixtures, with conductances of approximately 30–40 pS, consistent with data for syringopeptin 22B provided by Hutchison and Gross [39]. Dalla Serra et al. [28] estimated that syringopeptin 25A forms conducting complexes comprising at least four monomers and creates ion pores with a radius of about 1.2 nm.

**Figure 4:**
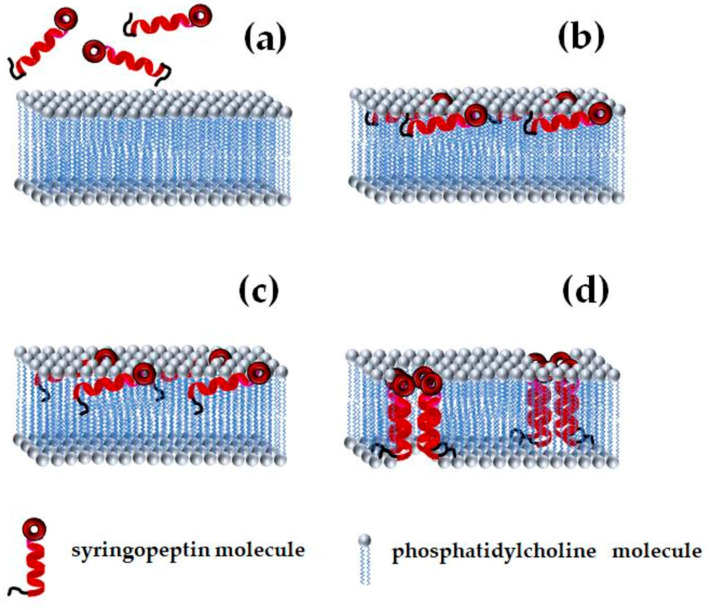
The suggested sequence for channel formation by syringopeptins includes the following steps: (**a**) the insertion of lipopeptide molecules into the lipid membrane; (**b**) the arrangement of the folded syringopeptin monomer within the membrane; (**c**) the alignment of the unfolded hydrophobic region of the lipopeptide with the lipid tails; and (**d**) the aggregation leading to the creation of a barrel-stave pore.

Functional studies of syringopeptin 25A channel kinetics show strong similarity to syringomycin E channels: predominantly anion-selective, voltage-dependent gating with two conductance states differing 4-fold [28,40]. Voltage-dependent channel opening and closing have also been described for syringopeptin 22A, syringomycin E, and syringotoxin B [25,28,37,41,42]. These gating behaviors likely result from conformational changes during transitions between open and closed states, driven by the movement of the charged CLP moiety within the electric field. Negative membrane potentials may favor channel opening by attracting the positively charged CLPs and extending the acylated hydrophobic peptide chain to fully span the membrane’s hydrophobic core [28]. Carpaneto et al. [40] proposed a multi-step channel formation mechanism involving monomer partitioning into the membrane, alignment of the unfolded hydrophobic peptide segment with lipid tails, and subsequent oligomerization to form barrel-stave-type pores. An exemplary scheme of syringopeptin insertion and formation of ion-permeable pores is shown in Figure 4.

### 2.5. Tolaasins

The tolaasin group includes tolaasins I–II, tolaasins A–E, tolaasin F, and sessilin [136,137,138,139]. These CLPs are produced by *P. tolaasii*, the causative agent of brown blotch disease in mushrooms. Their structures were determined by mass spectrometry, ^1^H NMR spectroscopy, and amino acid sequencing [140,141]. Each tolaasin contains 18 amino acids, with the *N*-terminus acylated by a (3R)-hydroxy fatty acid moiety; for example, tolaasin I contains 3R-hydroxy octanoic acid (3R-OH C8:0) (Figure 3). The C-terminal region cyclizes via an ester bond to a side-chain hydroxyl group. Unusual amino acids such as *Hse*, *Dab*, and *Dha* occur in tolaasins but are absent in other *Pseudomonas* CLPs (Figure 3). Several minor metabolite variants, tolaasins A–E, are structural homologs of tolaasin I. Tolaasin A differs by having an *N*-terminal 5-pentadioic acid (adipic acid, 5-COOH C5:0) instead of 3-hydroxy octanoic acid. Tolaasins B and D differ from tolaasin I by replacement of *Ile_15_* with *Val_15_* or *Ile_15_*, respectively. Tolaasin E exhibits two modifications: *Ile_15_* and *Hse_16_* are replaced by *Leu_15_* and *Gly_16_*. The linear homolog tolaasin C lacks the ester cyclization bond. Sessilin, produced by *P. sessilinigenes* CMR12a from cocoyam rhizosphere [139], differs from tolaasin I by *Glu_6_* replacing *Ser_6_*. Sessilin was effective in planta and in vitro against the oomycete pathogen *Pythium myriotylum*, which causes cocoyam root rot disease [142]; however, there is no current report on the antimicrobial activity of pure sessilin.

Using transmission electron microscopy, Cole et al. [143] showed that *P. tolaasii* acts on the fungal plasma membrane. Ferrarini et al. [144] reported that both tolaasin and sessilin induce cell leakage in fungal pathogens, with tolaasin I causing significantly greater leakage. The hemolytic activity of tolaasin was discovered by Rainey et al. [141] and Hutchison et al. [145]. The maximal effect on erythrocytes was at pH 6.5–7.0, with acidic or alkaline pH reducing lytic activity. Divalent metal ions inhibited hemolysis, suggesting selectivity of tolaasin-induced membrane conductance and implicating the formation of ion-permeable aggregates within the membrane [141,145].

Electrophysiological studies demonstrated channel formation by tolaasin in model membranes. Addition of 0.3 μM tolaasin to one side of phosphatidylethanolamine bilayers produced current fluctuations of ~0.7 pA at +20 mV, corresponding to CLP channel opening and closing [41]. Tolaasin-induced conductance showed voltage dependence, increasing markedly at positive transmembrane potentials—a finding later confirmed by Cho et al. [42]. Cho et al. identified two types of ion channels formed by tolaasin I in membranes composed of equimolar PS and PE: the predominant type 1 channels with ~5 pA current amplitude, and less frequent type 2 channels with amplitudes of 7–12 pA at 40 mV, with an approximate occurrence ratio of 4:1 (type 1/type 2) (Table 1). Both channels were cation-selective [41]. Rainey et al. [141] and Lo Cantore et al. [35] reported differences in tolaasin I pore dimensions, estimating functional pore radii of 0.9 ± 0.1 nm and 2.0 ± 0.4 nm, respectively; such discrepancies may relate to toxin concentration differences used in their studies.

Investigations of tolaasin’s secondary structure by Jourdan et al. [146] revealed that the amphipathic *N*-terminal α-helix penetrates deeply into the lipid bilayer core, while the hydrophilic, charged lactone ring remains near the membrane surface. Coraiola et al. [119] further confirmed significant insertion of tolaasin into the membrane’s hydrophobic core, suggesting deep anchoring beyond mere surface binding. Given tolaasin’s relatively small size, a single molecule is unlikely to form a full ion pore. Jo et al. [43], employing in silico modeling based on NMR data, proposed that tolaasin I channels consist of oligomers of approximately eight monomers. Coraiola et al. [119] proposed a barrel-stave model of pore formation, where tolaasin monomers insert into the bilayer and oligomerize to form a channel-like structure. Steigenberger et al. [147] experimentally confirmed oligomeric pore formation by tolaasin II within a barrel-stave framework. Notably, membrane thickness influenced tolaasin II activity, with thicker bilayers requiring higher local toxin concentrations to reach permeability thresholds, likely because the CLP molecules are too short to span the entire membrane in thick bilayers.

Furthermore, Coraiola et al. [119] showed that tolaasin I’s membrane permeabilization potency depends on lipid composition: increasing sphingomyelin content enhances activity, whereas sterols attenuate it. This inhibitory effect was more pronounced with cholesterol-containing membranes than with those enriched in ergosterol.

### 2.6. Fuscopeptins

Fuscopeptins A and B are CLPs produced by *P. fuscovaginae*. Their primary structures suggest an action mechanism similar to the well-known syringopeptins synthesized by *P. syringae* pv. *syringae*. Using fast atom bombardment NMR spectroscopy, mass spectrometry, and chemical and enzymatic analyses, the peptide moiety was characterized as containing 19 amino acid residues, with the terminal carboxyl group forming a macrocyclic lactone ring via linkage to the hydroxyl group of an *aThr* residue [148]. This lactone ring comprises five residues, formed between the hydroxyl group of Thr_15_ and the C-terminal *Phe_19_*. Fuscopeptins A and B are homologs, differing only in their fatty acid moieties: fuscopeptin A is *N*-acylated with 3-hydroxyoctanoate, while fuscopeptin B contains 3-hydroxydecanoate at the *N*-terminus [148] (Figure 3). The biological activities of fuscopeptins are closely related to those of syringopeptins, including causing damage to plant tissues and exhibiting antifungal activity against *Geotrichum candidum* and *S. cerevisiae* [86,149]. Coraiola et al. [44] demonstrated that fuscopeptins induce hemolysis in red blood cells, with fuscopeptin B exhibiting approximately 3-fold greater lytic activity than fuscopeptin A, correlating with the longer hydrophobic fatty acid chain. Calcein-loaded large unilamellar vesicles of various lipid compositions showed increased dye release upon exposure to fuscopeptins. The peptide concentration required to induce 50% vesicle membrane permeabilization closely matched the concentration causing red blood cell lysis [44]. Dynamic light scattering studies supported the conclusion that fuscopeptin-induced permeabilization does not alter vesicle size, thus excluding a detergent-like mechanism of action. Planar lipid bilayer experiments confirmed pore formation by fuscopeptins. Addition of fuscopeptins on one side of a POPC bilayer elicited discrete step-like increases in ion current, indicative of single-channel openings and closings [44]. Fuscopeptin B formed channels at lower concentrations (3–10 nM) than fuscopeptin A (40 nM) (Table 1). Single-channel current amplitudes for fuscopeptin B were approximately −0.4 pA at −140 mV and +1.3 pA at +140 mV, comparable to cormycin A produced by *P. corrugata* [127] but smaller than those of syringomycin and syringopeptin groups (Table 1). Using an electrolyte gradient, Coraiola et al. [44] measured a reversal potential of about +13.5 mV for both fuscopeptins, corresponding to a cation-to-anion permeability ratio (P^+^/P^−^) of ~2.1, indicating weak cation selectivity. This selectivity may be influenced by the conformation of the fuscopeptins’ lactone ring and peptide moiety, particularly involving the cyclic peptide segment containing two Dab residues. Circular dichroism and NMR spectroscopy revealed that fuscopeptins are unstructured in aqueous solution but adopt an α-helical conformation in membrane-mimicking environments [150]. This finding aligns with observations for tolaasin, where a predominance of helical structure in lipid membranes facilitated pore formation via a barrel-stave mechanism [119].

### 2.7. Entolysins

Entolysins were first described in studies of the entomopathogenic properties of their producing bacterium, *P. entomophila* L48. While entolysins are required for the swarming motility of *P. entomophila*, they do not contribute to its virulence toward *Drosophila melanogaster* [151]. The structural characterization of entolysin A and B, extracted from *P. entomophila* L48, was initially performed by amino acid analysis, MALDI MS/MS mass spectrometry, and gas chromatography [151]. Analysis revealed the presence of four *Leu*, one *Ile*, glutamine/glutamate (*Glx*), *Ser*, and *Val* residues. Fatty acid analysis showed that both compounds contain a 3-hydroxydecanoic acid (3-OH C10:0) moiety [151]. Bode et al. [152] used labeling experiments with deuterated [^2^H_9_] Leu and tandem mass spectrometry to refine the peptide sequence of entolysin A. Subsequent NMR studies confirmed that the structure of entolysin A produced by *P. entomophila* COR5 is identical to that of the L48 strain [153]. More recently, the full structures of both entolysin homologs (entolysin A and B) were established by chemical synthesis and NMR spectroscopy [154]. Entolysins A and B share identical amino acid sequences but differ in the stereochemical configuration of the Ser residue at position 13.

Vallet-Gely et al. [151] reported that *P. entomophila* L48 damages epithelial cells in the gut of Drosophila, a key factor in its virulence. Investigations into hemolytic activity linked the membrane-damaging effects of *P. entomophila* to entolysin production. Muangkaew et al. [154] confirmed the membrane-permeabilizing potency of both entolysins using propidium iodide staining, demonstrating that entolysins A and B disrupt fungal membranes of *P. oryzae* VT5M1 at a concentration of 16 µM. Notably, entolysin B exhibited significantly greater permeabilizing activity than entolysin A against *P. oryzae* VT5M1 and *B. cinerea* R16. Currently, the literature lacks further data on the effects of entolysins on membrane ion permeability. However, quartz crystal microbalance experiments by Reder-Christ et al. [45] revealed that entolysin B possesses strong binding affinity to model lipid membranes composed of POPC, DOPG, and ergosterol in various molar ratios. Membrane insertion by entolysin B occurred relatively independently of membrane composition [45]. Overall, while significant advances have been made in characterizing the structures and membrane interactions of entolysins, more comprehensive studies on their impact on membrane ion permeability remain to be conducted.

## 3. Cyclic Lipopeptides from *Streptomyces* spp.

**Figure 5:**
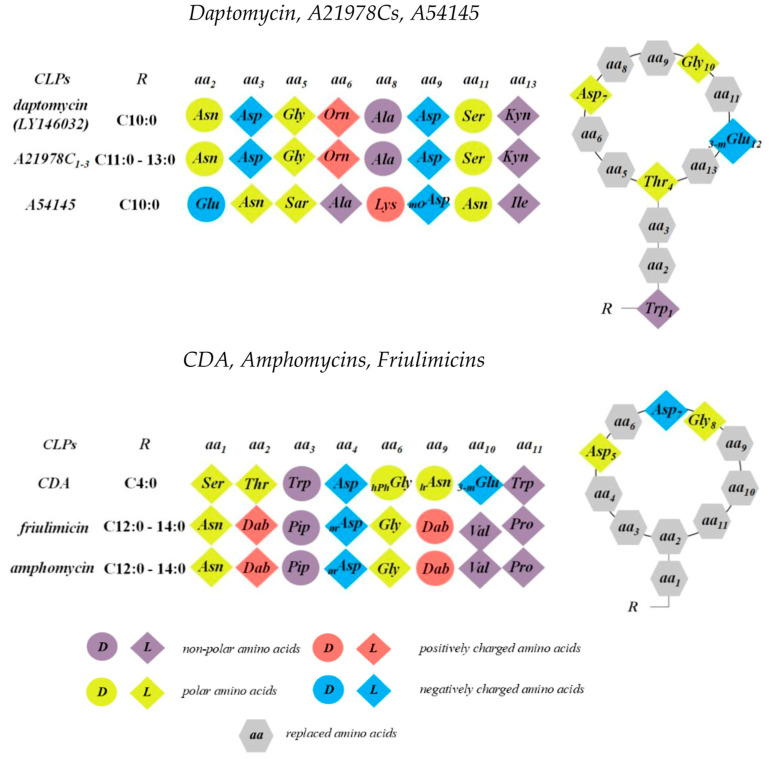
Chemical structures of pore-forming CLPs produced by *Streptomyces* spp. Abbreviations: *Sar*—sarcosine; *Orn*—ornithine; *mAsp*—*β*-methylaspartic acid; *mOAsp*—methoxyaspartic acid; *Kyn* —kynurenine; *3mGlu*—3-methylglutamic acid; *Dab*—2,4-diaminobutyric acid; *hPhGly*—4-hydroxyphenylglycine; *hAsn*—hydroxyasparagine.

One of the few CLPs used in clinical practice belongs to the class of calcium-dependent antibiotics (CDAs), which are produced by *Streptomyces* spp. The most well-known member of this group is daptomycin; however, several other compounds within this family also display potent antibacterial activity. These include depsipeptides isolated from *Streptomyces coelicolor* A3(2) and A54145 produced by *Streptomyces fradiei* [48,155]. Structurally, calcium-dependent CLPs share a highly conserved architecture characterized by specific arrangements of d-amino and achiral amino acids, notably featuring the conserved *Asp*-X-*Asp*-*Gly* fragment [156]. This motif is crucial for Ca^2+^ binding, which is essential for their antibacterial activity. Figure 5 illustrates the chemical structure of a nonapeptide with a cyclic head derived from *Streptomyces* spp. All members of the A21978C complex, including daptomycin, A54145, amphomycins, friulimicins, and other CDAs, are composed of cyclic decapeptides or decadepsipeptides with exocyclic tails of 1–3 amino acid residues, linked to a lipid tail.

Daptomycin exhibits strong antibacterial effects predominantly against Gram-positive bacteria. This includes methicillin-sensitive and methicillin-resistant strains of *Staphylococcus aureus*, coagulase-negative *Staphylococcus* spp. (including methicillin-sensitive and methicillin-resistant *Staphylococcus epidermidis*), various *Streptococcus* spp. such as *Streptococcus viridans*, and different isolates of *Streptococcus pneumoniae*, including penicillin- and macrolide-resistant forms. Moreover, daptomycin is active against vancomycin-sensitive and vancomycin-resistant enterococci, as well as *Enterococcus* isolates resistant to linezolid and quinupristin–dalfopristin. Friulimicin B and amphomycin, calcium-dependent CLPs related to daptomycin, exhibit similar antimicrobial spectra [15,157].

### 3.1. Daptomycin and the A21978C Complex

Daptomycin, a cyclic lipopeptide antibiotic, shares an identical peptide sequence with the A21978C complex produced by *Streptomyces roseosporus* [158]. This family of CLPs features conserved residues such as *Asp_7_*, *Asp_9_* (or modified *Asp_9_*), *Gly_10_*, followed by a d-amino acid at position 11, and d- or achiral residues at positions 5 and 8 [159] (Figure 5). The primary differences lie in the length of their fatty acyl chains: daptomycin has a straight-chain decanoyl side chain, whereas A21978C1, A21978C2, and A21978C3 bear 11-, 12-, and 13-carbon hydrocarbon tails, respectively (Figure 3). Subtle differences also exist in the three exocyclic residues, which include *Trp_1_*, a d-amino acid at position 2, and *Asn_3_* (or hydroxylated *Asn* (*hAsn_3_*)) [159].

Wale et al. [160] reported that daptomycin at 8 μg/mL induces severe morphological alterations in methicillin-sensitive and methicillin-resistant *S. aureus* and *Enterococcus faecalis*. Prolonged exposure leads to transformed cell surfaces and eventual degeneration into amorphous syncytial masses. At higher concentrations (25 μg/mL), daptomycin inhibits approximately 90% of amino acid and carbohydrate transport in *S. aureus*, suggesting significant membrane disruption [161]. Membrane permeabilization and depolarization by daptomycin are well documented across different pathogens. Using the electrochemical gradient probe TPP^+^, Alborn et al. [162] found that 100 µg/mL daptomycin reduced the membrane potential of *S. aureus* by ~50 mV. Silverman et al. [163] demonstrated membrane depolarization at lower concentrations (5 µg/mL) using the membrane potential-sensitive fluorescent probe DiSC_3_, correlating depolarization kinetics with bacterial viability. Similar depolarizing effects were observed in *B. megaterium* [161].

Daptomycin also triggers intracellular potassium leakage, indicating lipid bilayer disturbance. Allen et al. [164] employed potassium-specific electrodes to confirm membrane disruption in *B. megaterium* and *S. aureus*. Silverman et al. [163] used the potassium-sensitive fluorescent probe PBFI and observed immediate potassium release in *S. aureus* upon daptomycin addition, concomitant with decreased viability. Zhang et al. [165] demonstrated that 2.2 μM daptomycin causes significant potassium leakage from liposomes composed equimolarly of DMPC and DMPG. Kreutzberger et al. [166] similarly showed that this concentration induces ~36% collapse of giant unilamellar vesicles containing POPC and POPG in the presence of Ca^2+^. This membrane disruption likely results from stochastic CLP binding [166]. Lakey et al. [167] reported that daptomycin and A21978C homologs increase planar lipid bilayer conductance dose-dependently, reaching membrane destruction at 50–200 µg/mL. These conductance changes correlate well with in vivo lethal doses for homologs differing in acyl chain length. Seydlová et al. [46] confirmed daptomycin pore formation at 6.2 μM in planar lipid bilayers, observing ion channel conductances of 120 and 140 pS at 10 and 50 mV, respectively (Table 1). The lifetime and conductance of these pores increased with applied transmembrane voltage.

In solution, apo-daptomycin exists predominantly as a monomer adopting an extended conformation with bends at *Ala_8_*, *Gly_10_*/*Ser_11_*, *Asp_7_*, and *Asp_9_* [168,169]. Side chains predominantly extend outward, exposing to solvent, while the peptide backbone amides face inward. The *N*-terminal decanoyl chain exhibits flexible conformations [168].

Calcium binding is essential for daptomycin’s membrane activity. The *Asp*-X-*Asp*-*Gly* fragment binds Ca^2+^, which triggers conformational changes increasing amphipathicity and promoting membrane interaction [156,169]. Divalent cations vary in efficacy; Mn^2+^ replacement increases MIC 32-fold, while Mg^2+^, Cu^2+^, or Ni^2+^ raise MIC >64-fold compared to Ca^2+^ [170]. Calcium binding stoichiometry ranges from 1:1 to 2:3 (daptomycin:Ca^2+^), facilitating membrane binding or oligomerization [171,172,173]. Ho et al. [171] confirmed that aggregates of 14–16 daptomycin monomers form upon equimolar Ca^2+^ addition. Key Ca^2+^-binding sites involve *Asp_3_*, *Asp_7_*, *Asp_9_*, *MeGlu_12_*, *Trp_1_*, and *Kyn_13_* residues [168,169,171].

Daptomycin is believed to interact with Ca^2+^ through a two-step process. Both calcium-dependent phases are associated with fluorescent signals that reflect the movement of specific amino acid residues into less polar environments, indicating their introduction into the target bilayer [172]. The intrinsic fluorescence of daptomycin *Kyn_13_* demonstrated a minimal response both in the absence of calcium and at a concentration of 0.1 mM. However, it exhibited a rapid increase, nearing saturation at a concentration of 1 mM. Binding of the first Ca^2+^ increases the fluorescence of external labels attached to *Orn_6_* in daptomycin [172]. When the second Ca^2+^ binds, fluorescence emerges from the intrinsically fluorescent residues *Trp_1_* and *Kyn_13_*. Simultaneously, fluorescence from labeled residues 6 and 8 is quenched due to their mutual interactions. All four fluorophores are translocated to a more hydrophobic environment upon Ca^2+^ binding. This indicates that residues 1, 6, 8, and 13 play a role in daptomycin’s interactions with membranes, with residues 6 and 8 also involved in intersubunit interactions within the membrane-bound oligomer [172]. CLP oligomers likely assemble via π-π stacking interactions between the aromatic rings of *Trp_1_* and *Kyn_13_*, stabilized by Ca^2+^ ions mediating electrostatic repulsion [171].

**Figure 6:**
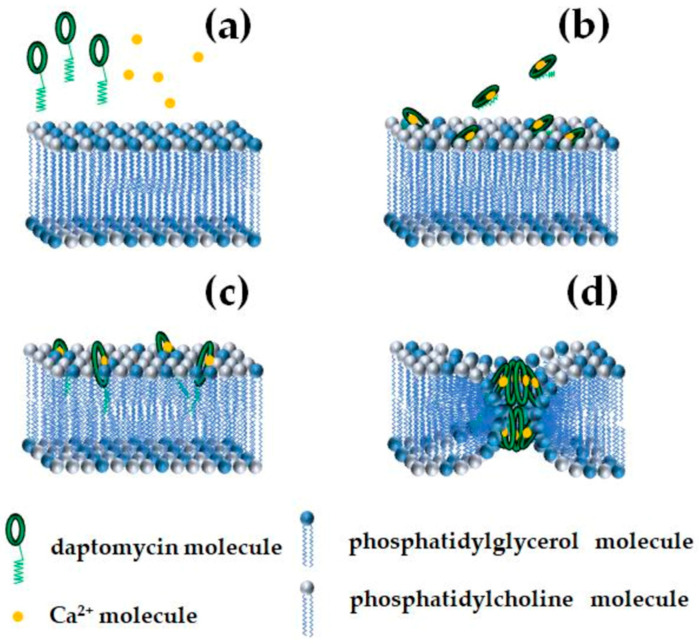
Hypothetical model of daptomycin membrane insertion into the lipid bilayer and pore formation: (**a**) presence of Ca^2+^-free daptomycin in the apo form in solution; (**b**) the binding of daptomycin to Ca^2+^ and its adsorption on the membrane. The decyl tail of the surface-bound daptomycin may be hidden and, as a consequence, CLP is unable to insert into the membrane; (**c**) the formation of a quaternary daptomycin–Ca^2+^–2 phosphatidylglycerol complex, where the decyl tail of the CLP is exposed and interacts with the acyl chains of the two phosphatidylglycerol molecules, facilitating CLP insertion; (**d**) translocation of a tetramer daptomycin through the membrane and production of the transmembrane octameric toroidal pores. The scheme was adapted from [165,166].

Oligomerization and membrane insertion depend on the presence of negatively charged PG lipids [169,174,175]. Phosphatidylglycerol induces deeper insertion and multimer formation. Using fluorescence of daptomycin–perylene conjugates, Muraih et al. [176] detected membrane insertion only in phosphatidylglycerol-containing membranes. Juhaniewicz-Dębinska et al. [177] reported that daptomycin causes moderate membrane fluidization, increasing average molecular area and decreasing compression modulus. Balleza et al. [178] also showed that daptomycin leads to an expansion effect on the lipid bilayer and promotes fluidization of PG-enriched lipid domains. Conversely, Machhua et al. [47] hypothesized that daptomycin’s decyl tail remains hidden on the membrane surface in a pre-inserting state and requires formation of a quaternary complex involving two phosphatidylglycerols to enable insertion and positive membrane curvature induction. The positive curvature strain leads to formation of “half-toroidal” pores composed of four daptomycin monomers, which fuse inner and outer dimples to create transmembrane octameric toroidal pores [47,179]. An exemplary scheme of daptomycin insertion and formation of ion-permeable pores is shown in Figure 6.

Cardiolipin, which is abundant in bacterial membranes, reduces daptomycin’s ability to translocate across membranes and form pores, despite its strong binding to membrane surfaces [180]. The conical geometry of cardiolipin increases lateral pressure in lipid tails, facilitating toroidal pore formation similarly to antimicrobial peptides like magainin [181]. Juhaniewicz-Dębinska et al. [177] demonstrated that daptomycin preferentially accumulates in one bilayer leaflet, increasing viscoelasticity locally and possibly affecting pore formation. Based on these observations, it is plausible that daptomycin may form pores via a mechanism analogous to that of syringomycin E. This mode involves the creation of CLP–lipid ion-permeable channels, characterized by distinct peptide and lipid “mouths.” However, the intrinsic negative spontaneous curvature of the monolayer can energetically hinder pore formation by increasing the cost of generating the lipid mouth’s positive curvature.

Membrane thickness also modulates daptomycin action: bilayers with shorter acyl chains (14 carbons) promote pore formation, whereas thicker membranes with 16 or 18 carbons allow only surface adsorption [182,183]. Increased lipid packing area similarly enhances insertion [184].

### 3.2. A54145

A54145 contains conserved residues similar to daptomycin, including *Asp_7_*, *Asp_9_* (or modified *Asp_9_*), *Gly_10_*, followed by a d-amino acid at position 11, and d- or achiral residues at positions 5 and 8. Additionally, it features sarcosine (*Sar*) at position 5, 3-methylglutamate (3-*MeGlu*) at position 4, and *Glu* at position 12 (Figure 5). This structural similarity suggests that A54145 may interact with lipid membranes through a mechanism analogous to daptomycin. Zhang et al. [185] investigated the interaction of A54145 with PG membranes and Ca^2+^ ions using fluorescence spectroscopy and isothermal titration calorimetry. The fluorescence intensity of pyrene-labeled A54145 increased in response to rising Ca^2+^ concentrations in two distinct phases. As Ca^2+^ concentration increased from 0 to beyond 1 mM, two sequential Ca^2+^-dependent transitions were observed, corresponding to the binding of two Ca^2+^ ions per molecule of A54145. Fluorescence data indicated that A54145 binds to the bilayer concomitant with the first Ca^2+^ binding event in solution, whereas oligomer formation was detectable only after the second Ca^2+^ ion bound. The absence of excimer emission suggested that at low micromolar concentrations, pyrene-labeled A54145 does not aggregate in solution. Site-specific fluorescence enhancements revealed that the first Ca^2+^ ion binds to *Lys_8_*, while the second ion interacts with *Trp_1_* and *Kyn_13_* residues. The translocation of all three fluorophores into a more hydrophobic environment upon calcium binding indicates that residues 1, 8, and 13 participate in bilayer interaction. Zhang et al. [185] proposed that binding of the first Ca^2+^ ion facilitates the membrane association of monomeric A54145, inducing the formation of a loosely bound state where acyl chains interact, but peptide regions—particularly residues 6 and 8—remain less engaged. Binding of the second Ca^2+^ ion ion triggers conversion to a tightly bound, functional oligomeric form [182]. This biphasic binding is consistent with prior models for daptomycin, wherein oligomers consist of four or eight monomers depending on whether they span one or both leaflets of the lipid bilayer [179]. Although no direct experimental evidence currently confirms transmembrane pore formation by A54145 CLPs, their structural and mechanistic resemblance to daptomycin suggests a potentially similar mode of lipid membrane disruption.

The daptomycin scaffold holds significant promise as a foundation for the development of new and effective antibiotics aimed at tackling the issue of global antimicrobial resistance [186].

### 3.3. Calcium-Dependent Antibiotics

The structure of CDAs features a macrocyclic peptide linked to an exocyclic *Ser* residue, which is lipidated at its *N*-terminus with (2S,3R)-3-propyloxirane-2-carboxylic acid (epoxyhexanoyl). CLPs from this group contain a combination of five unusual amino acids: d-*Trp_3_* (d-tryptophan), d-*HOPhGly_6_* (d-4-hydroxyphenylglycine), d-*HOAsn_9_* (d-erythro-hydroxyasparagine) or d-*POAsn_9_* (d-erythro-phosphoasparagine), *MeGlu_10_* (2S,3R-methylglutamic acid), and ∆*Trp_11_* (Z-dehydrotryptophan) (Figure 5). The structural diversity within the CDA class mainly arises from substitutions at positions 9–11. A hallmark that distinguishes CDAs from other CLPs is the highly conserved epoxyhexanoyl tail, present in different CDAs; in contrast, other Ca^2+^-dependent antibiotics exhibit considerable variation in tail length, saturation, or branching.

Lakey et al. [48] studied the effect of a CDA of unresolved structure on the conductance of planar lipid membranes composed of egg lecithin and cholesterol (2:1). In the presence of the CDA, they observed step-like current fluctuations with an amplitude of approximately 0.1 nS at a transmembrane voltage of 50 mV (~5 pA), consistent with stochastic opening and closing of ion-permeable channels (Table 1). The highest macroscopic membrane conductance was observed at ~16 mM Ca^2+^ concentration. Analysis of the membrane conductance dependence on CDA concentration indicated that 3–4 CLP monomers associate cooperatively to form a conducting pore. The channels were found to be cation-selective, implying the involvement of negatively charged residues lining the pore. Theoretical considerations proposed a channel diameter of approximately 0.6 nm [48].

To identify the lipid targets of CDA, Goodyear et al. [187] evaluated the effects of exogenously added phosphatidylglycerol, cardiolipin, phosphatidylethanolamine, phosphatidic acid, phosphatidylserine, and phosphatidylcholine on the MICs of CDA against *Bacillus subtilis*, alongside measuring fluorescence increases reflecting CLP insertion into liposomes. Liposomes containing cardiolipin exhibited the largest fluorescence increase compared to those with phosphatidylglycerol or other lipids, indicating deeper insertion of CDA into cardiolipin-rich membranes. Consistently, the MICs were significantly altered in the presence of cardiolipin, supporting its role as a primary lipid target. The authors proposed that CDA molecules simultaneously form two calcium-mediated bridges with the two phosphate groups of cardiolipin, enabling full membrane insertion. By contrast, phosphatidylglycerol, containing only one phosphate group, allows formation of only a single Ca^2+^ bridge, resulting in shallower membrane insertion of CDA [187].

A newly identified group of natural lipoglycopeptides, known as gausemycins, produced by *Streptomyces* sp. INA-Ac-5812, demonstrates significant effectiveness against Gram-positive bacteria [188,189,190]. Notably, gausemycin A has been found to rapidly compromise the membranes of *S. aureus* [189]. Research into resistance mechanisms suggests that alterations in the composition of membrane fatty acids occur alongside the development of resistance. Gausemycins interact with lipid bilayer in a Ca^2+^-dependent manner but require higher concentrations of Ca^2+^ to achieve optimal efficacy than daptomycin [190]. Gausemycins are able to form single ion channels, and this ability depends on the Ca^2+^ concentration and the lipid membrane composition [188,190]. Gausemycins constitute a structurally innovative class of lipoglycopeptide antibiotics, exhibiting distinctive membrane-targeting characteristics and significant antibacterial efficacy against Gram-positive pathogens, including resistant strains.

### 3.4. Amphomycin and Friulimicin

Amphomycins and friulimicins share a common macrocyclic peptide structure composed of 10 amino acids, cyclized via a bond between *Dab_2_* and *Pro_11_.* Their peptide rings contain several d- and non-proteinogenic amino acids, including *Thr_2_* and d-*allo*-2,3-*Dab_9_*, as well as d-*Pip_3_* (d-pipecolic acid) and *MeAsp_4_* (l-tero-3-methyl-aspartate) (Figure 5). These CLPs strongly interact with lipids and lipid-like molecules in cellular membranes, disrupting the integrity of the lipid matrix. Amphomycin’s interaction with calcium ions was studied using atomic force microscopy and circular dichroism spectroscopy, revealing a high specificity and affinity for certain divalent cations. In particular, the free energy of amphomycin monolayers decreased more significantly in the presence of calcium than with other tested cations. Oluwole et al. [191] investigated the effects of amphomycin and daptomycin on the binding of undecaprenyl phosphate (C55-P), an essential bacterial phospholipid, to its transbilayer transporter UptA, which plays a critical role in peptidoglycan synthesis. Unlike daptomycin, which does not disrupt the UptA-C55-P complex, amphomycin causes dissociation of this complex. This suggests that amphomycin may interfere with cell wall biosynthesis not only by binding free C55-P molecules but also through direct interaction with the UptA transporter [192].

Atomic force microscopy studies by Reder-Christ et al. [16] confirmed that calcium does not substantially affect the aggregation of friulimicin but is essential for its binding to model lipid membranes. Calcium-bound friulimicin strongly associates with phosphatidylcholine bilayers containing either 0.1 mol % undecaprenyl phosphate (C55-P) or dicetyl phosphate (DCP), a lipid analog presenting surface phosphate groups anchored by hexadecyl chains. In the absence of calcium, friulimicin showed no membrane binding. These findings highlight the critical role of the phosphate moiety of C55-P in mediating calcium-dependent CLP binding, with calcium acting as a bridge between the negatively charged peptide and the phosphate group. The lipid-like bactoprenol moiety of C55-P appears not to contribute specifically to binding, which is likely facilitated by non-specific interactions between the lipid tail of friulimicin and the membrane [16]. In addition to the membrane-binding and peptidoglycan-inhibition mechanisms outlined above, calcium-dependent CLPs such as amphomycins and friulimicins have also been reported to induce lipid membrane disorganization via formation of ion-conducting pores. This pore-forming activity represents an alternative or complementary mode of antimicrobial action widely discussed in the literature.

## 4. Cyclic Lipopeptides from *Bacillus* spp.

*Bacillus* and *Paenibacillus* spp. are well-known rich sources of a wide range of structurally diverse antimicrobials. The most studied representatives of CLPs produced by these genera include surfactins, iturins, fengycins, and polymyxins. These compounds have been extensively characterized for several decades and represent a potential gold mine of antibiotic candidates. Their chemical structures, biosynthetic gene clusters, antimicrobial activities, potential applications, and mechanisms of membrane disruption have been detailed in excellent comprehensive reviews [2,193,194].

Structurally, CLPs from *Bacillus* and *Paenibacillus* spp. consist of short oligopeptides linked to linear or branched fatty acids [195]. The cyclization mechanisms during biosynthesis of surfactins and iturins utilize the β-hydroxy or β-amino functionalities of fatty acids to form macrolactone and macrolactam rings, respectively [196]. Antibiotics from the octapeptin, fusaricidin, and paenibacterin groups are cyclic, cationic CLPs containing the non-proteogenic positively charged amino acid *Dab* [193].

**Figure 7:**
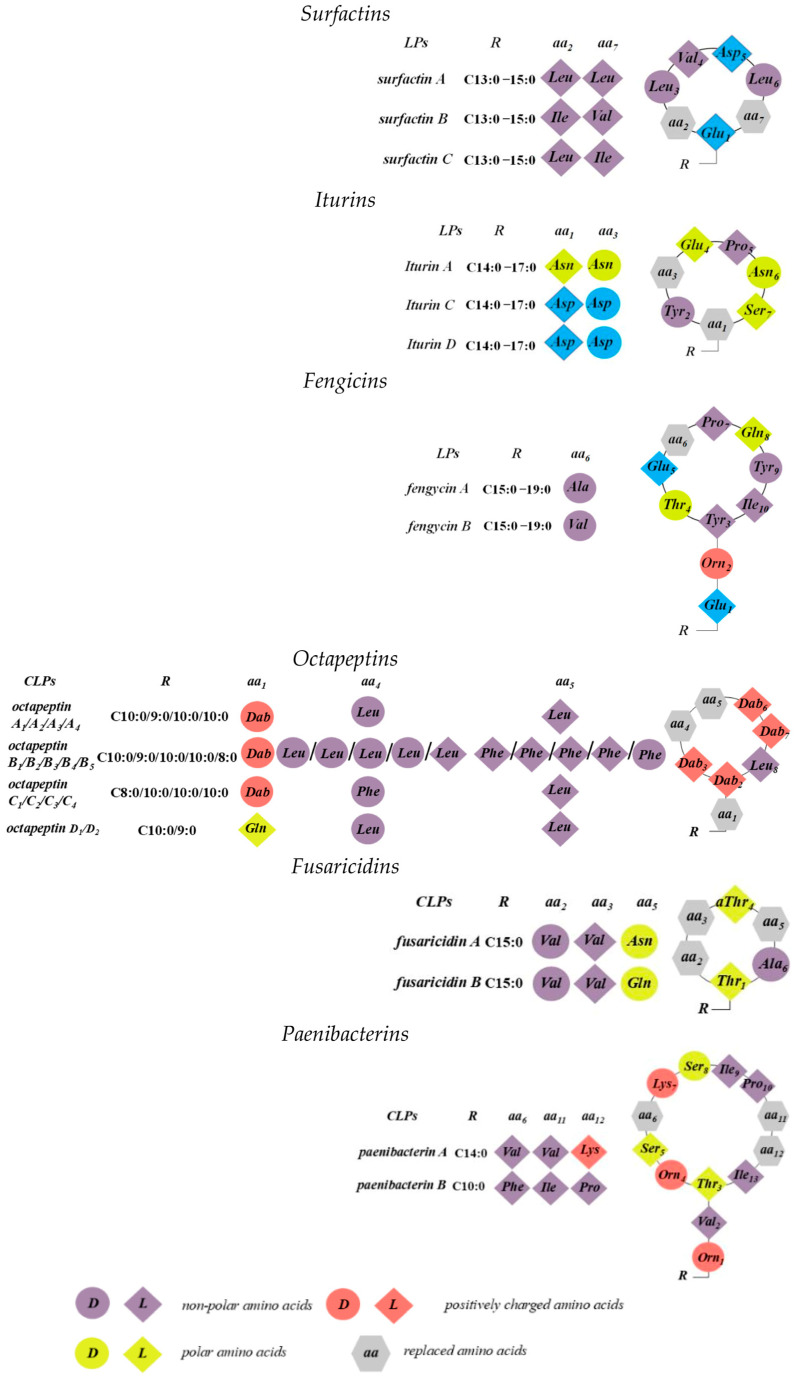
Chemical structures of pore-forming CLPs produced by *Bacillus* spp. *Abbreviations*: *Dab*—2,4-diaminobutyric acid; *Orn*—ornithine.

Octapeptins exhibit activity against common Gram-negative pathogens such as *E. coli*, *P. aeruginosa*, *K. pneumoniae*, and *A. baumannii*, though their potency is approximately 8- to 32-fold lower than that of polymyxins (which have MICs of 0.25–0.5 µg/mL) [194]. Unlike polymyxins, octapeptins also display moderate activity against Gram-positive bacteria. Moreover, they demonstrate antifungal activity. For example, octapeptin C_4_ was inactive against the fungi *Candida albicans*, *C. glabrata*, *C. parapsilosis*, and *Aspergillus fumigatus* (>100 µg/mL), but showed notable activity against *Cryptococcus gattii* (3 µg/mL) and several strains of *Cryptococcus neoformans* (1.5–6.2 µg/mL) [197]. Paenibacterin antibiotics exhibit potency against both Gram-negative and Gram-positive pathogens, including antibiotic-resistant strains of *E. coli*, *P. aeruginosa*, *A. baumannii*, *K. pneumoniae*, *S. aureus*, and *E. faecalis*. Importantly, paenibacterin shows relatively low cytotoxicity against human kidney cell lines (ATCC CRL-2190), with a 50% inhibitory concentration (*IC_50_*) ≥ 109 µg/mL, suggesting it as a promising candidate for new drug development [198]. Figure 7 illustrates the chemical structure of a nonapeptide with a cyclic head derived from from *Bacillus* spp.

### 4.1. Surfactins, Iturins, and Fengicins

The surfactin, iturin, and fengycin groups consist of distinct compounds that share peptides of the same length but differ in amino acid residues at specific positions. Each group includes several homologs, varying in the length and isomerism of their fatty acid chains, resulting in significant structural diversity [199]. Both surfactins and fengycins feature macrolactone rings. In surfactin, ring closure occurs between the β-hydroxyl fatty acid and the peptide’s C-terminus. Conversely, in fengycin, the peptide ring forms an ester bond between the *Tyr_3_* and the C-terminal residue, creating an internal ring analogous to many CLPs found in *Pseudomonas* spp. Similar to surfactins, the fatty acid chain of iturins is involved in cyclization; however, due to the presence of a β-amino group, an amide bond forms with the C-terminal group, resulting in a macrolactam structure [200]. Surfactin family compounds vary by amino acid identity at positions 2, 4, and 7, fatty acid chain length, and structural conformation (Figure 7) [201]. Fengycin exists primarily as two variants, fengycin A and B, differentiated by the amino acid at position 6 [202]. The peptide moiety is conjugated to a β-hydroxyl fatty acid chain (C12–C16), which may exhibit linear, *iso*, or *anteiso* branching. In all cases, the heptapeptide is linked to a β-amino fatty acid chain of variable length (C14–C17). The third family includes fengycins A and B, sometimes termed plipastatins when *Tyr_9_* adopts d-configuration. These decapeptides are attached to β-hydroxyl fatty acid chains (C14–C18), which can be linear, *iso*, or *anteiso* and may be saturated or unsaturated.

It was found that surfactin and iturin caused lysis of protoplasts prepared from *B. megaterium* and *Micrococcus luteus,* respectively [203,204]. The protoplast-degrading activity of surfactin was neutralized by the addition of phospholipids [203]. Disruption of viral lipid membranes and partial disruption of the capsid by surfactin was observed by electron microscopy [205]. Vollenbroich et al. [206] showed that surfactin affects the membranes of contaminating *Mycoplasma hyorhinis* and *Mycoplasma orale* using electron microscopy. The authors suggested that surfactin disrupts the plasma membrane, which is its main site of action. It caused membrane permeability and, at higher concentrations, led to complete membrane destruction and rupture of the mycoplasma. Fengycin-induced morphological changes such as swelling, twisting, or emptying of the hyphae have been detected in several fungi [207]. Furthermore, surfactin and fengycin exhibited strong and moderate hemolytic activity, respectively [205,207].

Surfactins, iturins, and fengycins bind to membranes via electrostatic and hydrophobic interactions and can disrupt lipid membranes depending on lipopeptide concentration due to micelle and pore formation. Detailed discussion of their incorporation into lipid bilayers, distribution between inner and outer leaflets, consequent lipid disorganization, and membrane permeabilization due to different modes of action is presented in exhaustive reviews [208,209,210,211].

Both surfactin and iturin A induce current fluctuations indicative of ion-permeable pores in lipid membranes. Surfactin-induced current fluctuations span a broader range compared to iturin A. In glyceromonooleate–decane bilayers, the conductance of iturin A-induced pores in 1 M KCl ranged from 6 to 30 pS, whereas surfactin pore amplitudes ranged from 10 to 140 pS [49,51]. In egg phosphatidylcholine bilayers, channel conductances for iturin A ranged from 7 to 50 pS [49]. Ostroumova et al. [50] demonstrated that multilevel conductance of surfactin pores is due to oligomerization of CLP molecules. According to Sheppard et al. [49], surfactin channels demonstrate pronounced cation selectivity, which is associated with two negatively charged amino acid residues in the molecule. Surfactin channels with higher current amplitude have lower cation selectivity [50], while only slight anion selectivity was found for iturin A [51,52,200]. Furthermore, the increase in macroscopic conductance with iturin A concentration, like surfactin, yielded a slope close to 2 [49,51,52].

Addition of fengycin to one side of a bilayer consisting of POPC/POPE/POPG/ergosterol at 0.1–0.5 μM leads to step-like current fluctuations of varying conductance in the pA range. Among channels induced by fengycin, single pores characterized by 1.5-fold higher conductance at positive transmembrane voltages than at negative ones predominate. Analysis of macroscopic current dependence on lipopeptide concentration suggests that fengycin dimers are involved in forming conductive subunits. Fengycin’s ability to form ion-conducting pores does not depend on the shape of membrane lipids but significantly correlates with the presence of negatively charged lipids, likely due to pore-forming properties depending on its membrane conformation [53].

### 4.2. Octapeptins

Octapeptins are a family of lipooctapeptide antibiotics with a broad antimicrobial spectrum, most of which were identified approximately 40 years ago [14,193,212,213,214,215]. This group consists of 18 related CLPs secreted by strains of *Bacillus circulans* and *Paenibacillus tianmuensis*, and they may be considered a subclass of polymyxins, sharing a similar cyclic heptapeptide ring with a truncated linear peptide tail [193] (Figure 7). Specifically, octapeptins possess a single exocyclic d-*Dab_8_* (or d-*Ser_8_*) residue instead of the l-*Dab_8_*-l*-Thr_9_*-l*-Dab_10_* tripeptide found in polymyxins, resulting in a reduction of the overall positive charge from five to four. Structural variation in octapeptins is reflected primarily in modifications of the *N*-terminal β-hydroxy fatty acid and/or the chirality of amino acids at positions 1, 4, and 5, leading to their classification into subgroups: octapeptins A, B, C, and D (Figure 7). Octapeptin A lacks *Phe* in its peptide structure, octapeptin B contains one *Phe_5_*, and octapeptin C is characterized by a *Phe_4_*. Octapeptin D differs by having *Ser* instead of *Dab_1_*, in addition to the absence of *Phe*. The peptide sequence is cyclized via a d-amino acid at position 1 and an *N*-terminal β-hydroxy fatty acyl chain. The length of this *N*-terminal hydroxyl-fatty acyl group varies from C8 to C10 among octapeptins and can be either non-branched (3-(R)-hydroxydecanoic acid) or branched ((R)-3-hydroxy-(S)-6-methyloctanoyl, 3-(R)-hydroxy-8-methyl-nonanoic acid, or 3-(R)-hydroxy-8(S)-methyldecanoic acid).

Using neutron reflectometry, Han et al. [216] investigated the interaction of octapeptin A3 with an asymmetric outer membrane model of resistant *P. aeruginosa*, where the outer leaflet consists of arabinose-modified lipid A and the inner leaflet of DPPC. Addition of octapeptin A3 at 1 µg/mL caused significant alterations in the neutron reflectometry profile. A substantial volume fraction of octapeptin A3 is localized within the hydrophobic acyl chains of both DPPC and lipid A. Octapeptins, like polymyxins, also undergo oligomerization. Isothermal titration calorimetry revealed a higher stoichiometry and binding affinity of octapeptin A3 for lipopolysaccharide compared to polymyxins. The binding stoichiometry for octapeptin A3 was approximately 3:1 (octapeptin A3/lipopolysaccharide), whereas colistin showed about 1.5:1 stoichiometry. Han et al. [216] suggested that complex formation with *P. aeruginosa* lipopolysaccharide involves both ionic and hydrophobic interactions.

Octapeptins affect lipid bilayers similarly to polymyxins. Swanson et al. [217] characterized the interaction of octapeptins with bacterial membranes and phospholipids via electron spin resonance, showing that the fatty acid chain of octapeptin inserts into the hydrophobic region of phospholipid bilayers and binds directionally to PG or PC. Later, Qian et al. [197] and Velkov et al. [194] reported that treating *P. aeruginosa*, *E. coli*, and *A. baumannii* cells loaded with the fluorescent dye *N*-phenylnaphthylamine with varying concentrations of battacin (octapeptin B5) and octapeptin C4 increased fluorescence due to bacterial outer membrane disruption. The potency of battacin and octapeptin C4 in releasing the dye was comparable to that of polymyxin B. Both compounds induced dose-dependent membrane depolarization in *P. aeruginosa* and *E. coli* [194]. The authors proposed that the correlation between antibiotic concentration and membrane depolarization effects reflects damage to the cytoplasmic membrane.

Bacterial cell permeabilization by octapeptin CLPs was further corroborated using surface plasmon resonance and leakage assays from lipid vesicles. The affinity of octapeptin C4 to model membranes was compared in the presence and absence of lipid A. Octapeptin C4 bound lipid A-free membranes but inserted significantly more into lipid A-containing membranes. Additionally, octapeptin C4 caused slight leakage of carboxyfluorescein from lipid vesicles composed of a neutral POPC and negatively charged POPG mixture (4:1 molar ratio) without lipid A [194]. This indicates that lipid A is required for octapeptins’ membrane-disruptive effects. Hartmann et al. suggested that the acyl chain of polymyxin B, a close structural analogue of octapeptins, penetrates the hydrophobic core of the bilayer, while charged residues anchor the peptide ring near the polar headgroup region, positioning the ring at the membrane surface [218,219]. Approximately six polymyxin B molecules form transmembrane pores with conductance amplitudes ranging from 1 to 5 pA in lipopolysaccharide-enriched bilayers (Table 1) [54].

### 4.3. Fusaricidins

Fusaricidins are lipid-modified non-ribosomal cyclic hexadepsipeptides composed of four d-amino acids and two l-amino acids (Figure 7). All members contain a *Thr* residue linked via the *N*-terminus to a unique 15-guanidino-3-hydroxypentadecanoic acid side chain. This distinctive ω-functionalized lipid moiety is essential for the antibiotic activity of fusaricidins and enables their selective targeting of bacterial cells through interactions with phospholipid membranes [220]. Three amino acids (*Thr*, d-*allo*-*Thr*, and d-*Ala*) are conserved across fusaricidins, which are often isolated in pairs differing by a single amino acid substitution (*Asp* replacing *Glu*). Several fusaricidins have been isolated from strains of *Paenibacillus polymyxa* [221,222]. Fusaricidins primarily exhibit antimicrobial activity against Gram-positive bacteria and a broad spectrum of fungi, including the plant pathogen *Leptosphaeria maculans*, responsible for blackleg disease in *Brassica crops* [223,224,225].

Recent studies reported that fusaricidins induce ultrastructural changes in *Phytophthora capsici* hyphae [226]. Scanning and transmission electron microscopy revealed collapsed and shrunken hyphal structures upon fusaricidin treatment, with irregular cell walls and disordered or missing organelles. Cell membrane integrity assessments via electrical conductivity, malondialdehyde content, and propidium iodide staining showed that low fusaricidin concentrations (0.5, 1, and 2 mg/mL) minimally affected hyphal electrical conductance [226]. However, at higher concentrations (5 to 10 mg/mL), conductance increased from 67 to 78%, indicating enhanced membrane permeabilization. Transcriptomic analysis (RNA-Seq) further supported that fusaricidin disrupts plasma membrane integrity, energy metabolism, transmembrane transport, and signal transduction in *P. capsici*. These results align with previous findings that fusaricidin disrupts *B. subtilis* membranes, impairing protein and nucleic acid biosynthesis at early stages of treatment [227].

Electrophysiological studies demonstrated that the addition of fusaricidins A and B at 0.6 μM to membrane-bathing solutions induced discrete step-like current fluctuations with amplitudes near 30 pA (Table 1). The transmembrane current increased roughly 6-fold with rising CLP concentration, indicating cooperative binding involving approximately six antibiotic molecules per pore formation in soybean phosphatidylcholine membranes [55]. Pore size in the inner mitochondrial membrane was probed using polyethylene glycols of varying molecular weights, revealing that fusaricidins A and B (11.8 μM) formed pores permeable to molecules averaging 750 Da [56]. This corresponds to a channel radius in the range of 0.69 to 0.89 nm.

### 4.4. Paenibacterin

Paenibacterin is an antimicrobial compound produced by *Paenibacillus thiaminolyticus* (the soil bacterium OSY-SE). The molecule consists of an 11-residue macrocyclic ring linked via an ester bond between the side chain of *Thr_3_* and the α-carboxyl group of *Ile_13_*, with an appended exocyclic dipeptide attached to a 15-carbon acyl tail [198,228] (Figure 7). Paenibacterin contains four basic residues, including *Orn* and *Lys*, which confer a cationic character under physiological conditions. The charged amino acid residues, together with two polar *Ser* residues, constitute the molecule hydrophilic portion. In contrast, the *N*-terminal lipid acyl chain and four aliphatic amino acids (*Val_6_*, *Ile_9_*, *Val_11_*, and *Ile_13_*) on one side of the β-sheet structure contribute to the molecule’s hydrophobicity [228]. This amphiphilic and cationic nature is thought to underlie paenibacterin’s broad-spectrum antimicrobial activity, primarily through electrostatic binding to anionic components of bacterial cell membranes, leading to disruption of membrane integrity and cell death [198]. Huang et al. [198] reported that paenibacterin at 32 µg/mL caused significant disturbance of bilayer potential in *E. coli* and *S. aureus*.

Additionally, treatment with paenibacterin induced concentration-dependent K^+^ leakage from these bacterial cells, indicating loss of cytoplasmic membrane integrity. Membrane permeability alterations were further confirmed using fluorescent dyes SYTO-9 and propidium iodide, which stain nucleic acids upon membrane compromise. Consequently, the paenibacterin bactericidal action is attributed to outer membrane disruption in Gram-negative bacteria and to damage of the cytoplasmic membrane in both Gram-negative and Gram-positive species. To date, there is no information regarding the ability of paenibacterin to form transmembrane pores.

## 5. Cyclic Lipopeptides from Cyanobacteria

Cyanobacterial CLPs share certain structural similarities with those produced by other bacterial groups [229]. Their peptide rings vary in size, ranging from as few as four amino acid residues, as seen in anabaenollysins, up to fourteen residues, such as in maleamides [230,231,232]. The lipid chain can be attached as a side group to an exocyclic amino acid via a single peptide bond (e.g., in cyanopeptolins or hassallidines) or incorporated directly into the cyclic core through two peptide bonds or a peptide and ester bond, forming macrolactam and macrolactone rings, respectively (e.g., minutissamides and others) [233,234,235,236]. Cyanobacterial CLPs often contain unusual and rare amino acids. Some are modified proteogenic residues such as hydroxyserine (*hSer*), hydrophenylalanine (*hPhe*), β-methylaspartate (β-*Me*-*Asp*), hydroxyproline (OH-*Pro*), hydroxyleucine (OH-*Leu*), hydroxyasparagine (OH-*Asn*), and valine-based β-amino-2-methylbutanoic acid. Others are unique to cyanobacteria, including p-amino-6-hydroxy-2-piperidone (*Ahopip*) found in cyanopeptolins and 2-(β-amino-5-oxotetrahydrofuran-2-yl)-2-hydroxyacetic acid (*AOFHa*) present in anabaenollysins. Notably, cyanobacterial CLPs are enriched in neutral amino acids such as *Val*, *Leu*, *Ile*, *Dha*, *Gln*, or *Asn*, whereas basic amino acids are rare, with *Arg* present in some cyanopeptolins.

The fatty acid moieties in cyclic cyanobacterial CLPs typically range from C5 to C18 in length. These fatty acids often contain unsaturation and/or multiple substituents, including halogenation, methylation, and hydroxylation. Incorporation of the fatty acid is enabled through hydroxylation or amination, typically at the β-carbon or another carbon, to facilitate bond formation. In some cases, hydroxylated fatty acids are further substituted with saccharides or amino acids [237,238].

**Figure 8:**
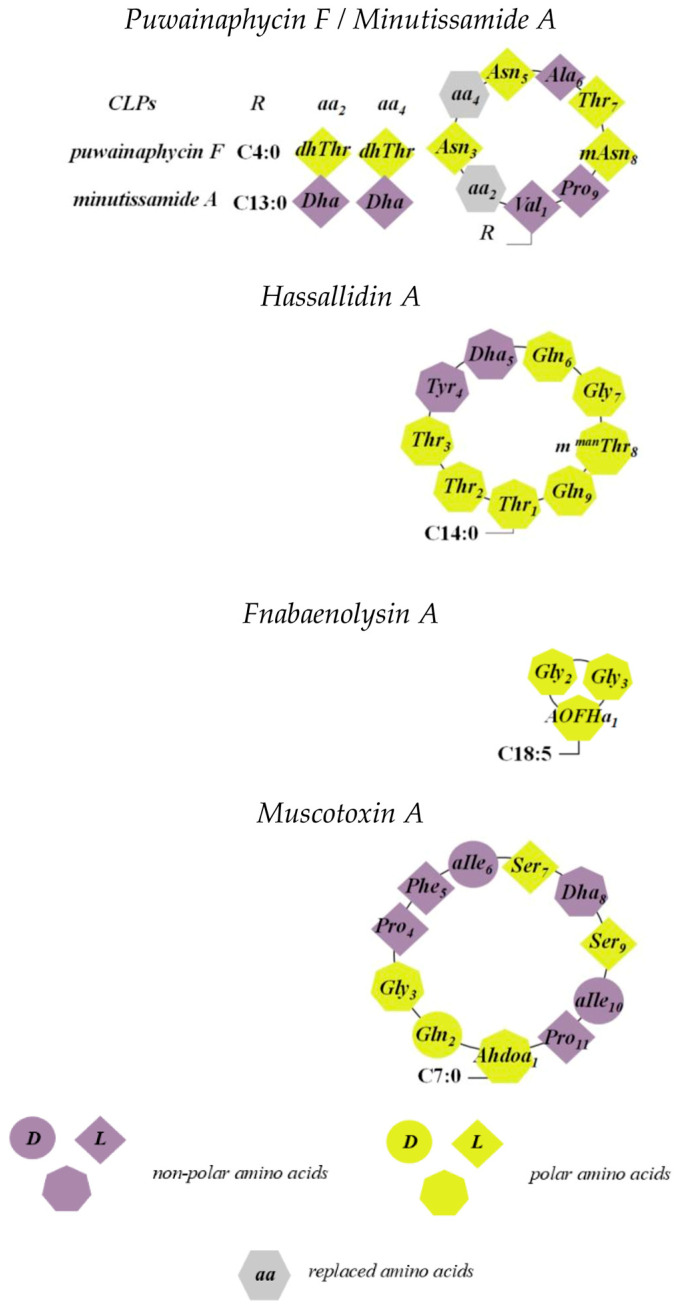
Chemical structures of pore-forming CLPs produced by cyanobacteria. *Abbreviations*: *mAsn*—*N*-methyl asparagine; *dhThr*—dehydrothreonine; *m ^man^Thr*—mannosyl-O-threonine; *AOFHa*—2-(3-amino-5-oxotetrahydrofuran-2-yl)-2-hydroxyacetic acid; *Ahdoa*—3-amino-2-hydroxydecanoic acid; *Dha*—2,3-dehydro-2-aminobutyric acid.

Puwainaphycins and minutissamides demonstrate diverse biological activities such as cardiovascular effects, antiproliferative properties, and antifungal action. Puwainaphycins exhibit greater activity against *C. glabrata* and *S. cerevisiae*, as measured by increased propidium iodide uptake. Their MICs were 3 to 129 times lower than those of minutissamides. The plant pathogen *Alternaria alternata* and the facultative human pathogen *A. fumigatus* showed high susceptibility to both compounds. Hassallidines display antifungal activity against opportunistic human fungi including *Candida* spp., *Aspergillus* spp., *Fusarium* spp., and *Penicillium* spp. [234,235,237,239,240]. Anabaenollysins exhibit cytolytic activity against various mammalian cell lines [241] and antifungal effects against *C. albicans* [242]. Pathogenic bacteria such as *S. aureus*, *Streptococcus sanguinis*, *P. aeroginosa*, and opportunistic pathogen *E. coli* are resistant to muscotoxins. Broad antifungal screening of these cyclic lipopeptides revealed strong lytic activity against *A. alternata*, *Monographella cucumerina*, and *A. fumigatus*. Figure 8 illustrates the chemical structure of a nonapeptide cyclic head representative of cyanobacterial CLPs.

### 5.1. Puwainaphycins and Minutissamides

Puwainaphycins A–G and minutissamids A–L are structurally analogous amphiphilic CLPs characterized by a nine-peptide ring cyclized to form a lactam ring between an amino acid residue and the amino group attached to the fatty acid moiety [243,244,245] (Figure 8). To date, twenty-one variants of these CLPs have been described, with structural diversity arising from differences in their peptide cores and substitutions of fatty acids of varying lengths (C10−C18). All reported chemical variants of puwainaphycins and minutissamides share the presence of a 2-hydroxy-3-amino-4-methyldodecanoic or 2-hydroxy-3-amino-4-methylhexadecanoic acid residue, as well as three non-standard amino acid residues: modified *N*-methylasparagine (*NMeAsn*) and two *Dha* fragments. Puwainaphycins and minutissamids have been isolated from cyanobacterial genera including *Cylindrospermum*, *Symplocastrum*, and *Anabaena*, exhibiting a wide range of biological activities, such as cardiovascular effects, antiproliferative action, and antifungal activity [56,244,245,246,247,248]. Both compound classes demonstrate strong lytic activity against eukaryotic cells. They exhibit comparable *IC_50_* values against human cervical cancer cells (HeLa), as measured by MTT assays [56]. Notably, puwainaphycin F exerted a stronger cytotoxic effect at higher concentrations (5–20 μM), reducing cell viability by 90–95%, whereas minutissamide A achieved approximately 70% inhibition within this concentration range. Hrouzek et al. [244] reported that the pronounced cytotoxicity of these CLPs is linked to their membrane-permeabilizing activity accompanied by calcium influx into the cytoplasm. Exposure of adenocarcinoma HeLa cells and primary human skin fibroblasts to puwainaphycins F and G at 1–10 μM caused decreased cell size and increased granulation. The compounds induced rapid intracellular calcium elevation, resulting in necrotic cell death upon prolonged exposure, suggesting a non-specific cytotoxic mechanism.

Additionally, Hájek et al. [57] compared the antifungal activities of puwainaphycins and minutissamides against a small panel of filamentous fungi. Puwainaphycins demonstrated greater membrane-permeabilizing potency than minutissamides against *C. glabrata* and *S. cerevisiae*, as evidenced by propidium iodide uptake. The MICs of puwainaphycins were 3 to 129 times lower than those of minutissamides. Both compounds displayed highest efficacy against the plant pathogen *Alternaria alternata* and the facultative human pathogen *A. fumigatus*. Significant differences in permeabilizing potency between puwainaphycins and minutissamides were also observed in planar model membranes composed of DOPC/DOPE (2:1) mixtures [57]. Puwainaphycin F induced ion-permeable pore formation at 10 μM, whereas minutissamide A required approximately twice this concentration to achieve similar activity (Table 1). Typical transmembrane current amplitudes were ~10 pA for minutissamide A and ~500 pA for puwainaphycin F. Furthermore, puwainaphycin F at 7 μM elicited leakage of carboxyfluorescein from liposomes composed of DOPC/DOPG (1:1) [249]. These observations highlight the structural and functional diversity within the puwainaphycin/minutissamide family and underscore the critical influence of fatty acid moiety variations on biological activity and cytotoxicity.

### 5.2. Hassallidins

Hassallidins are secondary metabolites first discovered in a strain of the genus *Hassallia* [234,235]. These CLPs have also been reported in some cyanobacterial genera, including *Anabaena*, *Aphanizomenon*, *Cylindrospermopsis*, *Nostoc*, *Planktothrix*, and *Tolypothrix* [234,239,250]. The peptide ring of hassallidins consists of eight amino acids, including an exocyclic amino acid, and is glycosylated with one to three sugar moieties [234,235,239,250] (Figure 8). Cyclization of the eight-membered peptide macrocycle occurs via an ester bond between the exocyclic Thr residue and a fatty acid moiety [234]. Hassallidins vary considerably in the length of their fatty acid chains (C14–C18) and glycosylation patterns, which occur at the Thr residues of the peptide ring as well as at the β-hydroxyl position of the modified fatty acid.

Hassallidins exhibit antifungal activity against opportunistic human pathogenic fungi, including *Candida, Aspergillus*, *Fusarium*, and *Penicillium* spp. [234,235,239,250]. Their bilayer-disrupting activity has been investigated in human myeloid leukemia cells, normal rat kidney cells, fungal cells, artificial membranes, and through *in silico* modeling. In studies by Neuhof et al. [235], the effect of hassallidin A on the ultrastructure of *C. albicans* was analyzed by electron microscopy, revealing membrane-surrounded fragments near the plasma membrane that were absent in untreated controls. Such structures likely represent accumulated secretory vesicles formed due to plasma membrane disruption and abnormal vesicle formation beneath the rupture sites as discribed for the echinocandin-like lipopeptide micafungin [251]. Treatment of human acute myeloid leukemia (MOLM-13) cells with hassallidin caused disruption of bilayer integrity leading to complete dissolution of the bilayer and exposure of organelles and the nucleus, as confirmed by transmission electron microscopy. The membrane activity of hassallidin was further assessed using liposomes composed of pure phospholipids or mixtures of phospholipids and cholesterol. When hassallidin was added to cholesterol-containing liposomes, it caused significant release of calcein, with nearly complete release occurring at 0.8 μM in a short time. In contrast, cholesterol-free liposomes exhibited only modest calcein release even at 30 μM hassallidin. Liposomes containing ergosterol were also highly sensitive to hassallidin [252]. In silico modeling corroborated these findings, highlighting a dependence of hassallidin’s membrane activity on the presence of cholesterol [252]. The acyl chain of hassallidin was observed to insert into PC bilayers only when cholesterol was present. Rapid insertion of the fatty acid moiety into cholesterol-rich membranes was associated with intercalation of a *Tyr_5_* ring into the polar region of the lipid bilayer. In the absence of cholesterol, hassallidin interacted only weakly with the PC bilayer surface. To date, there is no available information on the ability of hassallidins to form ion-permeable pores.

### 5.3. Anabaenolysins

Anabaenolysins are a distinctive class of CLPs produced by benthic strains of the cyanobacterial genus *Anabaena* inhabiting the Baltic Sea [21,230]. Their structure features a rare unsaturated β-amino fatty acid bearing a conjugated triene system and a small four-membered peptide ring composed of two proteinogenic amino acids and an unusual moiety, 2-(3-amino-5-oxotetrahydrofuran-2-yl)-2-hydroxyacetic acid (Figure 8). Multiple chemical variants of anabaenolysins exist, distinguished primarily by variations in the length (C16–C19) and the specific nature of the conjugated trienic β-amino fatty acid. Functionally, anabaenolysins exhibit cytolytic activity against various mammalian cell lines [241] and antifungal effects, including activity against *C. albicans* [21]. LC_50_ values for anabaenolysin A and B range from 4.4 to 14 µM and 3.7 to 17 µM, respectively, across different mammalian cell types [230]. Investigations of rat hepatocyte surface morphology using phase-contrast and differential interference microscopy suggested permeabilization of the outer plasma membrane by anabaenolysins. Oftedal et al. [241] reported that anabaenolysins function as amphipathic biodetergents, with anabaenolysin A showing higher hemolytic potency than digitonin.

**Figure 9:**
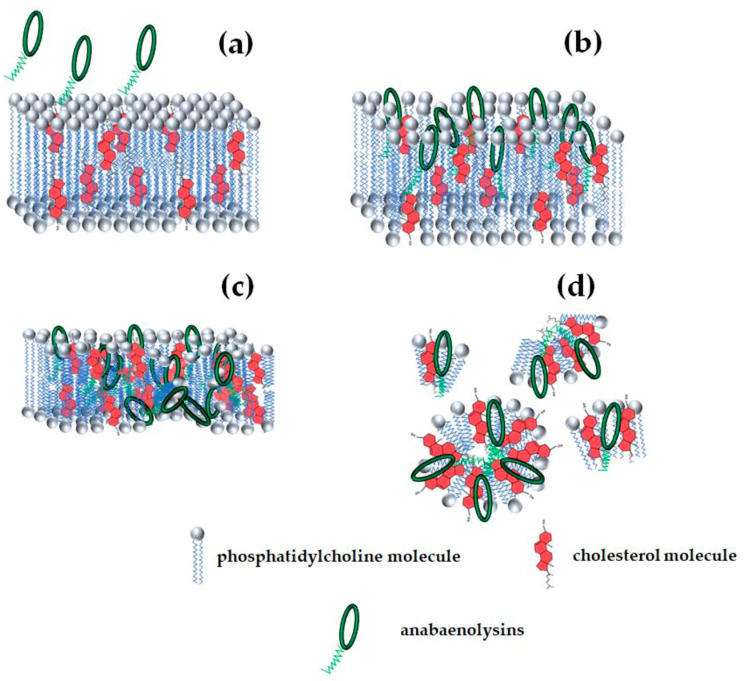
The proposed mechanism for the interaction of anabaenolysin A with lipid membranes can be described as follows based on scientific understanding and relevant studies: (**a**) the initial attachment of the lipopeptide to the lipid bilayer; (**b**) the integration of anabaenolysin A into the membrane, causing localized disruption of the bilayer, which results in alterations in permeability; (**c**) the uneven distribution of anabaenolysin A between the inner and outer leaflets; (**d**) lipid disorganization and changes in membrane permeability through a detergent-like action.

Studies conducted on lipid membranes derived from NB4 leukemia cells, hepatocytes, and model bilayers, augmented by in silico modeling, further characterized their mode of action. Exposure to anabaenolysin A caused a reduction in cell size and induced propidium iodide uptake in NB4 leukemia cells, indicative of compromised membrane integrity [241]. Transmission electron microscopy revealed extensive intracellular content loss upon treatment. Experiments assessing calcein release from phosphatidylcholine liposomes demonstrated greater permeabilization efficiency of cholesterol-enriched membranes compared to cholesterol-free ones. This suggests a specific affinity of anabaenolysins for cholesterol, posited to destabilize membrane structure. Additionally, anabaenolysins may induce segregation of membrane components, altering membrane organization. Comparative analyses of their lytic capacity in liposomes composed of soybean phosphatidylcholine versus hydrogenated phosphatidylcholine suggested a strong ability to intercalate between lipid acyl chains and cholesterol within the membrane’s hydrophobic core. Collectively, these findings indicate that anabaenolysins target cellular membranes containing sterols, conferring potent effects suited for selective disruption of surface and non-mitochondrial organelle membranes. While the geometry of anabaenolysins suggests a potential for transmembrane pore formation, no direct experimental evidence currently exists to confirm such pore-forming activity. An exemplary scheme of anabaenolysin A insertion and formation of ion-permeable pores is shown in Figure 9.

### 5.4. Muscotoxins

Muscotoxins A–C are CLPs isolated from the filamentous cyanobacterium *Desmonostoc muscorum* that disrupt mammalian cell membranes and induce necrotic cell death [253]. The muscotoxin peptide ring comprises 11 amino acid residues, including a non-branched 3-amino-2-hydroxydecanoic acid (5-*OH Ahdoa*) with a short C10 aliphatic chain; the non-proteinogenic *Dhb*; two unusual d-amino acid isoforms, d-*Gln* and d-*allo*-*Ile*; and γ-methylproline (γ-*MePro*) in muscotoxin B, which is replaced by *Pro* in muscotoxins A and C (Figure 8). Muscotoxins exhibit weak antibacterial activity against Gram-positive *B. subtilis* with a MIC value of 37.5 µg/mL [253]. Pathogenic bacteria such as *S. aureus*, *S. sanguinis*, *P. aeroginosa,* and opportunistic pathogen *E. coli* are resistant to muscotoxins. Broad screening of the antifungal activity of these lipopeptides revealed strong lytic effects on *A. alternata*, *Monographella cucumerina*, and *A. fumigatus,* with MIC values of 0.58, 2.34, and 2.34 µg/mL, respectively. Similar to other cyanobacterial CLPs, muscotoxins display marked cytotoxicity towards human cells. The potency of muscotoxin A is comparable to that of anabaenolysin CLPs from benthic *Anabaena cyanobacteria,* with reported LC_50_ values ranging from 3.7 to 17 µM against multiple cell types (exposure time unreported), and to puwainaphycins, which show LC_50_ values of 2.15–2.19 µM against HeLa cells after 24 h of exposure [249].

Tomek et al. [249] demonstrated that muscotoxin A rapidly perturbs lipid membranes and induces the flux of small ions into HeLa cells, triggering cell death. The permeabilizing action of muscotoxin A was assessed using synthetic liposomes comprising an equimolar mixture of DOPC and DOPG, monitored by fluorescent dye release. At 25 µM, muscotoxin A prompted significant carboxyfluorescein leakage, indicative of membrane disruption. Incorporation of cholesterol and sphingomyelin into the liposomes significantly modulated bilayer fluidity near the gel–liquid crystalline phase transition temperature [249]. The authors suggested that muscotoxin A’s membrane-disruptive activity depends more on bilayer fluidity and organization than on specific interactions with lipid components such as cholesterol. Overall, these findings support that cyanobacterial muscotoxins disrupt mammalian cell membranes by increasing membrane permeability. To date, no evidence has been reported for their ability to form transmembrane pores.

## 6. Conclusions

CLPs are receiving increasing attention as a distinct class of compounds offering advantageous characteristics as antibiotics in comparison to small molecules and macromolecular antibodies. *Pseudomonas* spp. and *Bacilli* spp. are among the best-known natural producers of these compounds. However, accumulating evidence suggests that cyanobacteria are an equally rich and promising source of CLP antibiotics. While much remains to be understood about the mechanisms of action of various CLPs, several key properties have been identified. In particular, although CLPs vary in length, sequence, and amino acid composition, most possess an amphiphilic structure and generally exhibit membrane activity. CLPs bind to bacterial membranes and incorporate into the lipid bilayer through electrostatic interactions (due to the charged peptide moiety) and hydrophobic interactions (due to the lipid tail).

Another common feature of many CLPs is their ability to self-assemble at the target cell membrane. This assembly leads to membrane depolarization and/or leakage of intracellular components through the formation of oligomeric transmembrane pores or defects in lipid packing. The literature reports that CLPs assemble into aggregates composed of monomers ranging from 2 (e.g., iturin or fengycin) to 8 (e.g., tolaasin or daptomycin). Some aggregates form in solution, which facilitates delivery of higher concentrations of the agents to the target membrane. Typically, the ion-permeable aggregates generate current amplitudes in the picoampere range. Thus, CLPs are capable of forming pores according to generally accepted models of pore formation. Based on the structural features of the CLPs considered here, it can be assumed that agents with relatively large cyclic peptide rings and small external peptide segments are capable of forming toroidal pores. Examples include syringomycin E, viscosin, and daptomycin. A second group comprises CLPs with external peptide tails containing many amino acid residues, such as syringopeptins, tolaasins, and fuscopeptins. These agents form ion-permeable pores according to the barrel-stave model. For CLPs whose pore-forming capability has not been experimentally demonstrated, one may infer from the available data that they likely possess similar abilities.

Given the rise in antibiotic resistance and the increasing mortality from infections, new antimicrobial drugs are being developed with the goal of resisting resistance development over time. This often involves formulating combination antibiotics. Such combinations not only enhance efficacy by targeting multiple pathways simultaneously, but also reduce the likelihood of resistance emergence in pathogenic strains. The findings from this review may help broaden the spectrum of available therapeutic agents.

## Figures and Tables

**Table 1:** Charecteristics of pore-forming activity of cyclic lipopeptides CLPs produced by *Pseudomonas* spp., *Streptomyces* spp., *Bacillus* spp., and cyanobacteria.

CLPs	Lipid Composition	*C*, µM	The Amplitude of Ion-Permeable Pores	m	Reference
*Pseudomonas* spp					
syringomycins	DOPE/DOPS/DOPC/cholesterol (3/1.5/0.5/5)	0.4–1.6	1 pA at 80 mV	ND	[24]
	DOPS	0.8–4.1	0.7 pA at 100 mV	5–7	[25]
	DOPC	0.8–2.5	−0.8 pA at −100 mV; 1.2 pA at 100 mV	ND	[26]
	DPhPC	10	−1.5 pA at −180 mV	ND	[27]
	red blood cells	ND	ND	5–7	[27]
	DOPC/sterols (cholesterol, ergosterol, or stigmasterol) (1/1)	ND	ND	5–7	[28]
	DOPE/DOPS/sterols (cholesterol, ergosterol, or stigmasterol) (1/1/2)	2.5	−2.6 pA at −100 mV −12 pA at −200 mV	ND	[29]
syringotoxins	DOPC	7.9–14.1	−1.4 pA at −100 mV; 1.6 pA at 100 mV	ND	[26]
	phospholipids isolated from soybeans	0.7	2 pA at 10 mV	ND	[30]
	red blood cells	ND	ND	4–6	[27]
		ND	ND	2–6	[31]
syringostatins	DOPC	1.4–2.1	−1.4 pA at −100 mV; 1.9 pA at 100 mV	ND	[26]
	DOPE/DOPS (1/1)	0.6–0.9	0.9 pA at 150 mV	ND	[32]
pseudodesmins	DOPE/DOPG/CL (7/2/1)	3.7	ND	ND	[33]
	DOPE/DOPG (3/7)	0.17	ND	ND	[34]
viscosins	DOPE/DOPG/CL (7/2/1)	5.0	ND	ND	[33]
	DOPE/DOPG (3/7)	0.1	ND	ND	[34]
viscosinamides	DOPE/DOPG/CL (7/2/1)	3.6	ND	ND	[33]
WLIP	DOPE/DOPG/CL (7/2/1)	4.7	ND	ND	[33]
	red blood cells	ND	ND	6–10	[35]
cormycin A	DPhPC/cholesterol (7/3)	<0.05	−0.6 pA at −150 mV	6–8	[36]
syringopeptin 22A	DOPE/DOPS (1/1)	0.1	0.2 pA at 100 mV 0.3 pA at −100 mV	2–4	[37]
	DOPC	0.1	ND	ND	[38]
syringopeptin 22B	DOPE	2.3	1–1.5 pA at 80 mV	ND	[39]
syringopeptin 25A	DOPC/DOPE/DOPS (2/2/1)	0.004	−5.5 pA at −140 mV 4.1 pA at 140 mV	4–5	[28]
	DOPC/sterols (cholesterol, ergosterol, or stigmasterol) (1/1)	ND	ND	4–6	[28]
	asolectin	0.0125	2.2 pA at 140 mV −6.3 pA at −140 mV	ND	[40]
	DOPC	0.1	ND	ND	[38]
tolaasin I	POPE	0.3	5 pA at 20 mV	ND	[41]
	PS/PE (1/1)	0.0159	7–12 pA 40 mV	ND	[42]
	ND	ND	ND	6–8	[43]
	red blood cells	ND	ND	5–7	[35]
fuscopeptins A	POPC	0.040	ND	ND	[44]
fuscopeptins B	POPC	0.003–0.01	−0.4 pA at −140 mV 1.3 pA at 140 mV	ND	[44]
entolysin B	POPC/DOPG (9/1), POPC/DOPG/ergosterol (6/1/3; 4/1/5)	0.5	ND	ND	[45]
*Streptomyces* spp.					
daptomycin	DPhPG	6.2	5 pA at 100 mV	ND	[46]
	DMPC/DMPG (9/1)	ND	ND	8	[47]
calcium-dependent antibiotics	egg lecithin/cholesterol (2/1)	3 v %	5 pA at 50 mV	ND	[48]
*Bacillus* spp.					
surfactin	GMO	1.4	0.5–7 pA at 50 mV	2	[49]
	DPhPC	0.2–0.4	2–240 pA at 25 mV	ND	[50]
iturin	egg lecithin	0.0001	0.7–5 pA at 100 mV	ND	[51]
	GMO	ND	0.6–3 pA at 100 mV	2	[52]
fengycin	POPC/POPE/POPG/ergosterol (2/2/5/1)	0.0001–0.0005	1 pA at 150 mV	2–3	[53]
polymyxin B	DOPC/DOPG (1/1)	5	1–5 pA at 50 mV	2–3	[54]
	DOPC/DOPG/Kdo_2_-Lipid A (1/1/0.02)	1	1–5 pA at 50 mV	5–7	
fusaricidins A + B	azolectin	0.6	30 pA at 60 mV	ND	[55]
fusaricidins A + B	mitochondrial inner membrane	11.8	ND	ND	[56]
*Cyanobacteria*					
puwainaphycins F	DOPC/DOPE (2/1)	5	500 pA at 50 mV	ND	[57]
minutissamid A	DOPC/DOPE (2/1)	10	10 pA at 50 mV	ND	[57]

*C*—threshold CLP concentration required to observe single ion-permeable pores; m—the number of CLP molecules that participate in the formation of a transmembrane pore. Abbreviations: DOPE—1,2-dioleoyl-*sn*-glycero-3-phosphoethanolamine; DOPS—1,2-dioleoyl-*sn*-glycero-3-phosphoserine; DOPC—1,2-dioleoyl-*sn*-glycero-3-phosphocholine; DOPG—1,2-dioleoyl-*sn*-glycero-3-phosphoglycerol; DPhPC—1,2-diphytanyl-*sn*-glycero-3-phosphocholine; DPhPG—1,2-diphytanyl-*sn*-glycero-3-phosphoglycerol; POPE—1-palmitoyl-2-oleoyl-*sn*-glycero-3-phosphoethanolamine; POPC—1-palmitoyl-2-oleoyl-*sn*-glycero-3-phosphocholine; azolectin—L-α-phosphatidylcholine from soybean; GMO—glyceromonoolein; CL—cardiolipin; Kdo_2_-Lipid A—di [3-deoxy-d-manno-octulosonyl]-lipid A (ammonium salt). ND—the value of step-like amplitude was not determined.
